# Supplementary material for: Systems Genome: Coordinated Gene Activity Networks, Recurring Coordination Modules, and Genome Homeostasis in Developing Neurons
Source: Int J Mol Sci. 2024 May 22;25(11):5647. doi: 10.3390/ijms25115647 (PMC11171963; doi:10.3390/ijms25115647)
Supplement: Supplementary file 1 [file ijms-25-05647-s001.zip › ijms-2955710-SM.pdf]

Supplementary Materials for

Systems genome: coordinated gene activity networks, recurring coordination modules, and genome homeostasis in developing neurons

Siddhartha Dhiman *et al.*

*Corresponding author Email: mks4@buffalo.edu

This PDF file includes:

Supplementary Text

Figs. S1 to S11

Tables S1 to S3

1.1.1 Supplementary Text

1.1.2 Computational methods

A machine learning algorithm that draws inferences from datasets without labels was used to reveal potential hidden structures and patterns in data in an unbiased manner (Fig. 1). Specifically, *k*-means clustering was applied to the transitional fold change dataset. This algorithm partitions *n* observations of fold changes into *k* clusters, with each observation belonging to the cluster with the nearest mean *k* value. The value of *k* clusters was optimally deduced using Calinski–Harabasz evaluation. Prior to unsupervised clustering, the fold changes were converted to a logarithmic scale to linearize fold changes whereby a 1-fold change (or no change) and 2-fold change would correspond to 0 and 1 on the logarithmic scale, respectively.

1.1.3 Gene–gene coordination represented via correlations

Gene activities in three biological samples of NCCs or NPCs cultured and harvested independently [7] were correlated under each condition; correlated gene pairs were computed in a single condition. For this, the RNAseq data were first standardized with a z-score. This standardization centered and scaled RNAseq observations to have a mean of 0 and standard deviation of 1. When comparing NPCs and NCCs (example given in fig. S1C), the individual NPC conditions (C1, C2, and C3) or NCC conditions (N1, N2, and N3) were standardized across the entire column for a given gene. Following standardization, the pairwise Pearson correlation between each gene pair was computed for both conditions, as shown in fig. S1D. Variables *x* and *y* refer to the RNAseq observations of the two conditions in a comparison, and $\overline{x}$ and $\overline{y}$ are the means. This correlation was computed for every gene in a comparison, for each condition. In a comparison containing *n* number of genes, the number of pairwise correlations is represented by the equation:

$$nC_2 = \binom{N}{2} = \frac{n!}{2(n-2)!}$$

For RNA datasets from cells derived from four control individuals and four with schizophrenia [18], the correlations among four samples (control or schizophrenia) were computed separately across pairs of genes using the protocol described above.

The frequency distributions of Pearson coefficients (*r* from -1 to +1) in different groups were compared and statistically evaluated using χ^2 tests (Fig. 2 and 3; fig. S3). To identify *r* values that were different from random, the positive (>0 to +1) and negative (<0 to -1) *r* frequency data were fit to a β model (example shown in fig. S3A). The β distribution is a family of continuous probability distributions defined on the interval [0, 1] or (0, 1) in terms of two positive parameters (denoted α and β) that appear as exponents of the variable and its complement to 1, respectively, and control the shape of the distribution. The 95% critical threshold values (mean ±SD) in each analyzed group were identified and listed in fig. S3A.

Pearson cross-correlation (heat maps)

OriginLab software was used for graphical and statistical analyses. This program produced heat maps comparing cross-correlations between different groups of genes, including correlations between genes regulated or not during NPC→NCC transition, between neurodevelopmental genes and transcription factor genes, and between genes dysregulated or not in schizophrenia.

Cytoscape network analyses

For the network analyses of correlation pairs with *r* values >0.99, two layouts were used to show the visual differences between the different conditions.

In the first layout, Cytoscape [29] created a circular graph layout by using an algorithm that produces layouts that emphasize group and tree structures within a network. It partitions the network by analyzing its connectivity structure and arranges the partitions as separate circles (examples in Fig. 4 and fig. S7). Network analyses were performed on all the circular graphs to obtain the attributes listed below, which define the circular networks:

1. Total number of nodes (i.e., genes) present in the network.

2. Number of edges as the overall number of linkages between the nodes.

3. Average number of neighbors, which shows the average connectivity of a node in the network.

4. Network diameter, which is the maximum length of the shortest paths between two nodes; if a network is disconnected, its diameter is the maximum of all diameters of its connected components.

5. The shortest path length between two nodes.

6. Network density, which is a normalized version of the average number of neighbors[29] and a value between 0 and 1. This indicates how densely the network is populated with edges (self-loops and duplicated edges are ignored). A network with no edges that is solely isolated has a density of 0.

7. Clustering coefficient of a node *n* in an undirected network is $Cn = 2en/[kn(kn - 1)]$, where kn is the number of neighbors of *n* and en is the number of connected pairs between all neighbors of *n(41).* In directed networks, the definition is slightly different: $Cn = en/[kn(kn - 1)]$. In both cases, the clustering coefficient is a ratio, N/M, where N is the number of edges between the neighbors of *n*, and M is the maximum number of edges that could possibly exist between the neighbors of *n*. The clustering coefficient of a node is always a number between 0 and 1.

8. Connected components, which indicates the connectivity of a network: two nodes are connected if there is a path of edges between them. All the nodes that are connected pairwise form a connected component; a low number of connected components suggests stronger connectivity.

The second Cytoscape-based layout used in our study is the hierarchical clustered layout, which uses the algorithm for representing main direction or "flow" within a network[29] Nodes are placed in hierarchically arranged layers and the ordering of the nodes within each layer is chosen in such a way that minimizes the number of edge crossings. This algorithm is available by selecting Layout → Hierarchical Layout[29]. The results are shown on Fig. 5A–C.

FANMOD motif analysis

RNAseq data were used to determine whether nondirectional gene coordination in GANs involves a set of recurring coordination motifs. Network motifs were created using FANMOD, a tool for network motif detection[32]. This software accepts a network as input and detects network motifs as patterns that occur more often in the network than in random networks of the same size and with the same connectivity properties. The software accepts network data in the form of a list that details the interactions that occur between different genes, outputs the recurring network motifs, and depicts these motifs within the network. Given the limitations of the available computational power, motifs composed of 3–6 gene nodes were investigated initially.

RCM frequency statistical analyses (used in Fig. 7A,B and in fig. S9A,B)

The Shapiro–Wilk test was used to determine if the analyzed groups had normal distributions. Because the RCM frequency curves in NCC and NPC GANs were not normally distributed, the nonparametric Wilcoxon rank sum test was used. The analyses were conducted in MATLAB, with a P value of <0.05 considered significant.

Modeling RCMs as information-processing resistor (R), inductor (L), capacitor (C) circuit.

We analyzed the output of the R-L-C circuit as follows. The input in a series circuit is defined as a time-varying voltage:

$$V_{in} = V_R + V_L + V_C \ ,$$

The output of the circuit, V_{out} , can be represented as the voltage across the capacitor:

$$V_{out} = V_C \ ,$$

$$V_{in} = RC\frac{dV_c}{dt} + LC\frac{d^2V_c}{dt^2} + V_c.$$

To calculate $\frac{V_{out}}{Vc}$, we create a differential equation by using values of current and voltage drop across R, L, and C and solve for the output voltage as a function of these values and input. On solving, we get three cases:

Overdamped case: $\left(\frac{\mathrm{R}}{2\mathrm{L}}\right)^2 > \frac{1}{LC}$

In this case, the output signal loses some part of the input to the response. The slope of the output signal rise is smaller than the input signal (which would indicate a loss of information in an information network).

Critically damped case: $\left(\frac{R}{2L}\right)^2 = \frac{1}{LC}$

In this case, the output closely follows the input, no oscillations are caused, and no information is lost.

Underdamped case: $\left(\frac{R}{2L}\right)^2 < \frac{1}{LC}$

In this case, the output is underdamped, which means that for a small change in input, oscillations are induced in the output, and substantial noise is added to the information being transmitted.

We can describe the same circuit's transfer function as:

$$H(s) = K\frac{1}{(s-p_1)(s-p_2)},$$

where, $p_1 and\ p_2$ are poles of system, and

$$K = \frac{1}{LC},$$

$$\mathrm{p}_{1,2} = \omega_\mathrm{n} - \beta \mp \sqrt{(\beta^2 - 1)}),$$

$$\beta = \frac{R}{2}\sqrt{\frac{C}{L}},$$

$$\omega_n = \sqrt{\frac{1}{LC}},$$

and $\beta > 1$ is the overdamped case, $\beta = 1$ is the critically damped case, and $\beta < 1$ is the underdamped case.

Using this transfer function, we can calculate the transfer function of the network by cascading them as follows:

$$H(s_1, s_2 \dots) = H(s_1)H(s_2)\dots$$

Supplementary Figures

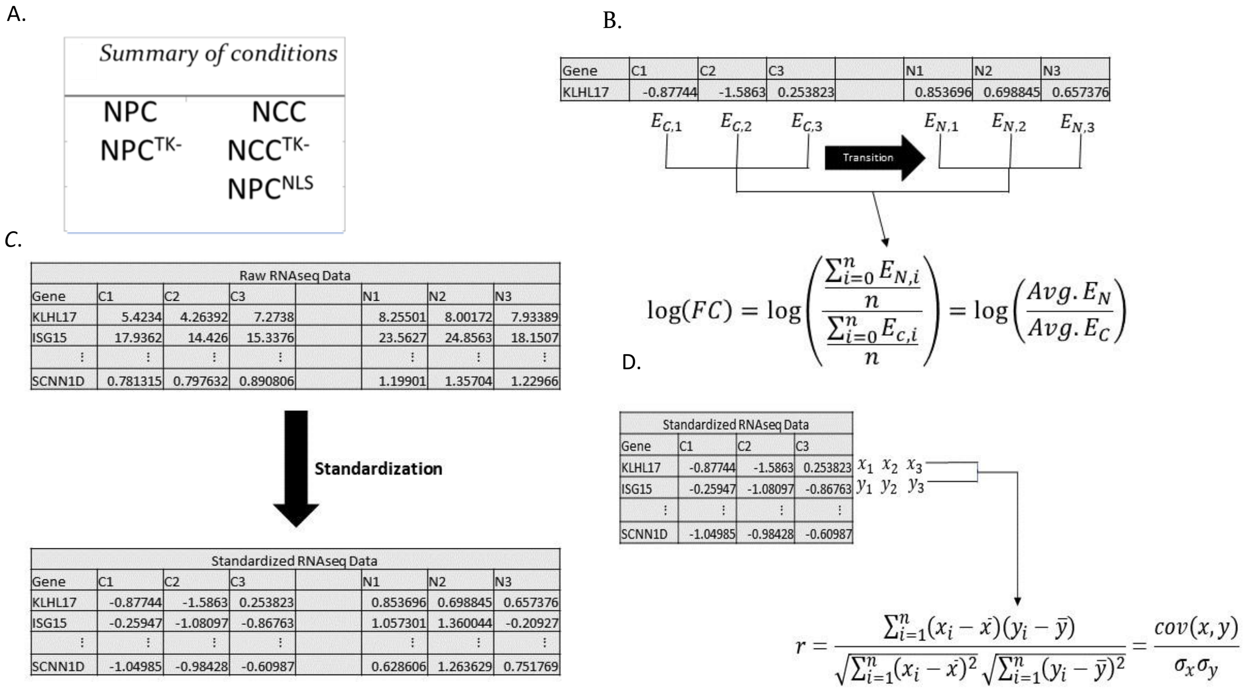

Fig. S1. Gene activity correlation analyses.

(**A**) Summary of cell conditions*(7,*: NPC, neural progenitor cells (nondifferentiated); NCC, neuronal committed cells (early differentiated neurons); NPC^{TK-}, NPC transfected with dominant negative nuclear FGFR1(SP-/NLS)(TK-); NCC^{TK-}, NCC differentiated from NPC transfected with dominant negative nuclear FGFR1(SP-/NLS)(TK-); NCC^{NLS}, NCC differentiated from NPC transfected with constitutively active nuclear FG FR1(SP-/NLS). (**B**) Calculation of logarithmic fold change of NCC (N1, N2, N3)/NPC (C1, C2, C3). (**C**) Standardization of RNAseq data [7]; the table on top shows the raw RNAseq readings, where C1, C2 ,and C3 are different NPC cultures and N1, N2, and N3 are different NCC cultures. The table below shows the result of z-score standardization. (**D**) Calculation of Pearson correlation coefficient (*r*) of standardized data.

A.

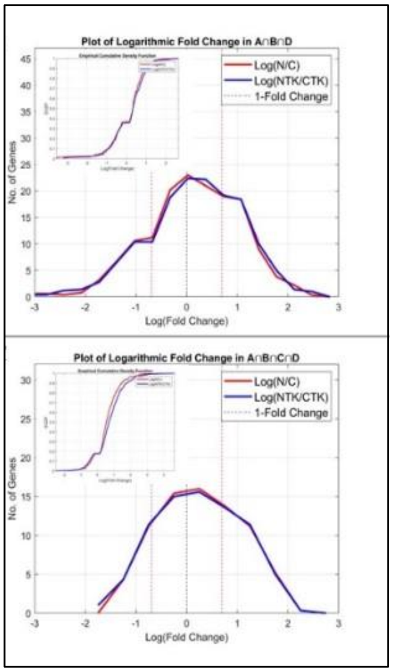
Plot of Logarithmic Fold Change in A∩B∩D
Log(N/C)
Log(NTK/CTK)
1-Fold Change
No. of Genes
Log(Fold Change)
Plot of Logarithmic Fold Change in A∩B∩C∩D
Log(N/C)
Log(NTK/CTK)
1-Fold Change
No. of Genes
Log(Fold Change)

B.

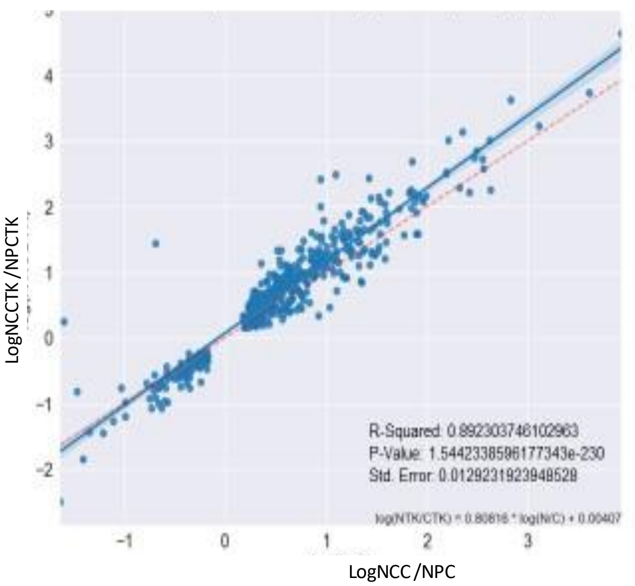
LogNCCTK/NPCTK
R-Squared: 0.892303746102963
P-Value: 1.5442338596177343e-230
Std. Error: 0.0129231923948528
log(NTK/CTK) = 0.80816 * log(N/C) + 0.00407
LogNCC/NPC

C.

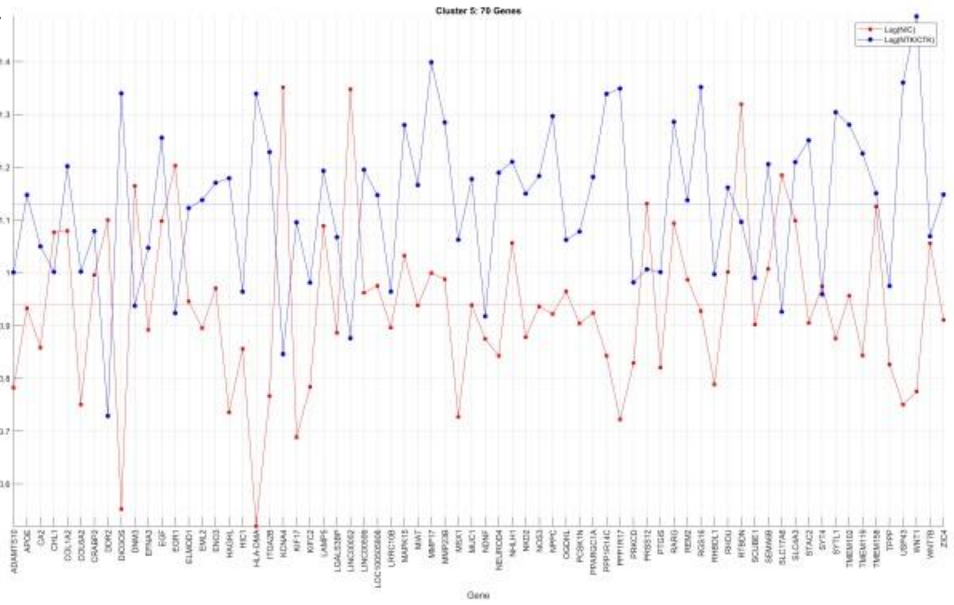
Cluster 5: 70 Genes
Log(N/C)
Log(NTK/CTK)
Fold Change
Gene

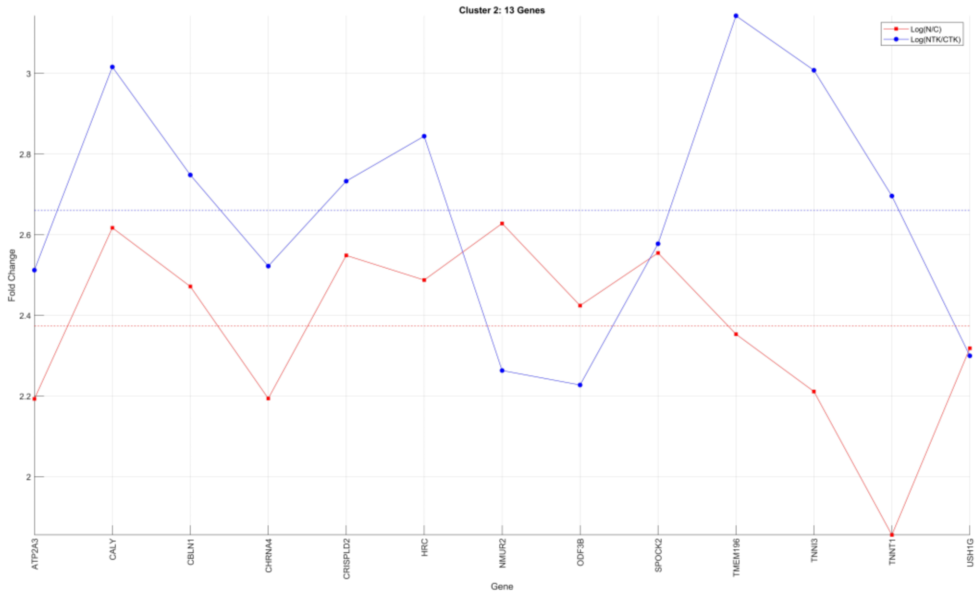
Cluster 2: 13 Genes
Log(N/C)
Log(NTK/CTK)
Fold Change
3
2.8
2.6
2.4
2.2
2
ATP2A3
CALY
CBLN1
CHRNA4
CRISPLD2
HRC
NMUR2
ODF3B
SPOCK2
TMEM196
TNNI3
TNNT1
USH1G
Gene

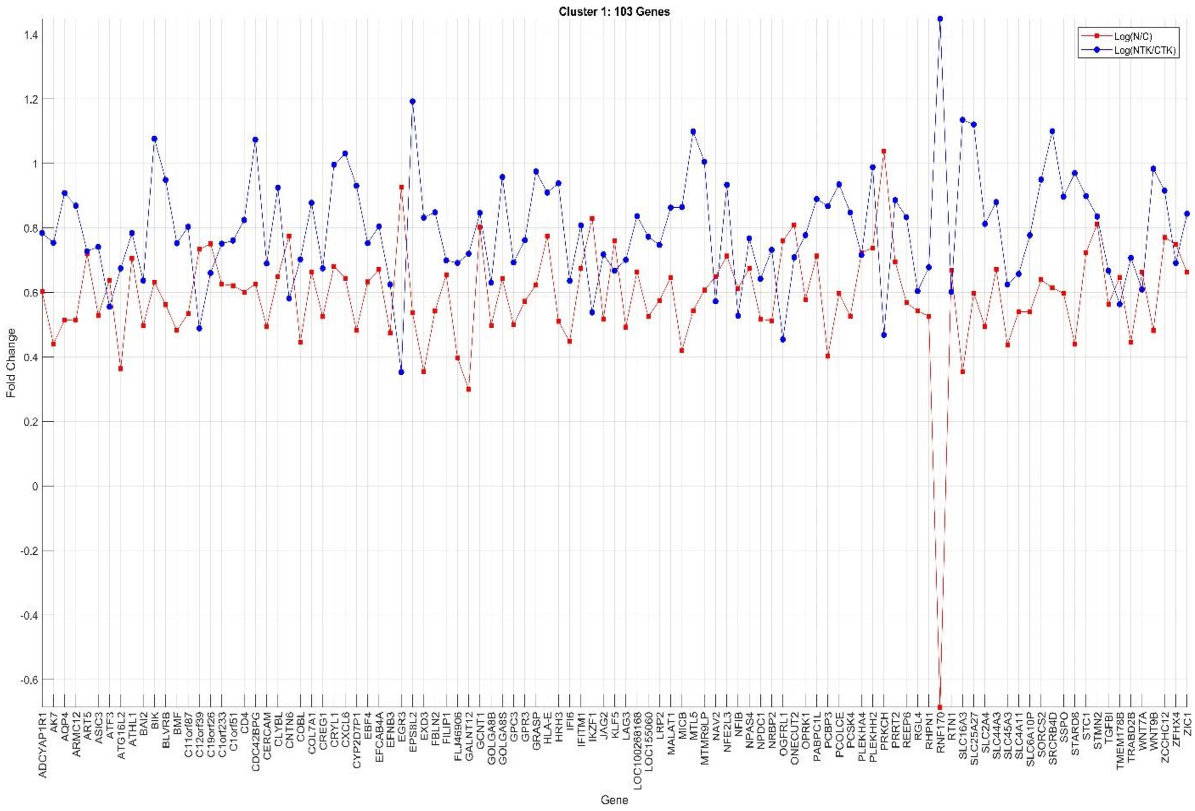
Cluster 1: 103 Genes
Log(N/C)
Log(NTK/CTK)
Fold Change
1.4
1.2
1
0.8
0.6
0.4
0.2
0
-0.2
-0.4
-0.6
ADCYAP1R1
AK7
AQP4
ARMC12
ART5
ASIC3
ATF3
ATG16L2
ATHL1
BAI2
BIK
BLVRB
BMF
C11orf87
C12orf39
C19orf26
C1orf233
C1orf51
CD4
CDC42BPG
CERCAM
CLYBL
CNTN6
COBL
COL7A1
CREG1
CRYL1
CXCL6
CYP2D7P1
EBF4
EFCAB4A
EFNB3
EGR3
EPS8L2
EXD3
FBLN2
FILIP1
FLJ46906
GALNT12
GCNT1
GOLGA8B
GOLGA8S
GPC3
GPR3
GRASP
HLA-E
HRH3
IFI6
IFITM1
IKZF1
JAG2
KLF5
LAG3
LOC100268168
LOC155060
LRP2
MALAT1
MICB
MTL5
MTMR9LP
NAV2
NFE2L3
NFIB
NPAS4
NPDC1
NRBP2
OGFRL1
ONECUT2
OPRK1
PABPC1L
PCBP3
PCOLCE
PCSK4
PLEKHA4
PLEKHH2
PRKCH
PRRT2
REEP6
RGL4
RHPN1
RNF170
RTN1
SLC16A3
SLC25A27
SLC2A4
SLC44A3
SLC45A3
SLC4A11
SLC6A10P
SORCS2
SRCRB4D
SSPO
STARD8
STC1
STMN2
TGFBI
TMEM178B
TRABD2B
WNT7A
WNT9B
ZCCHC12
ZFHX4
ZIC1
Gene

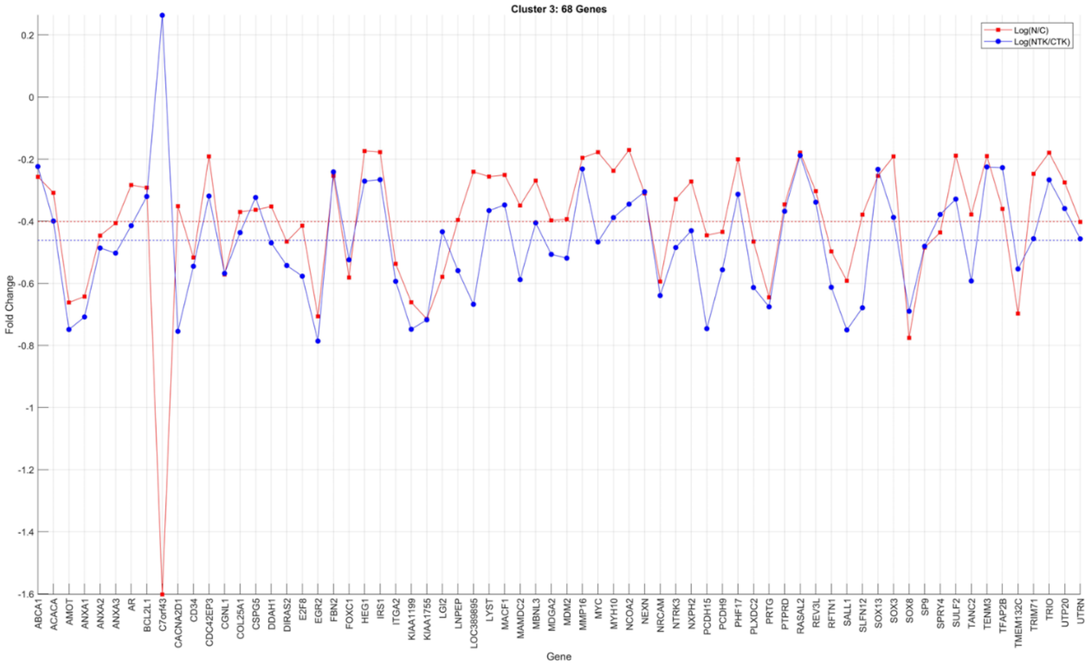

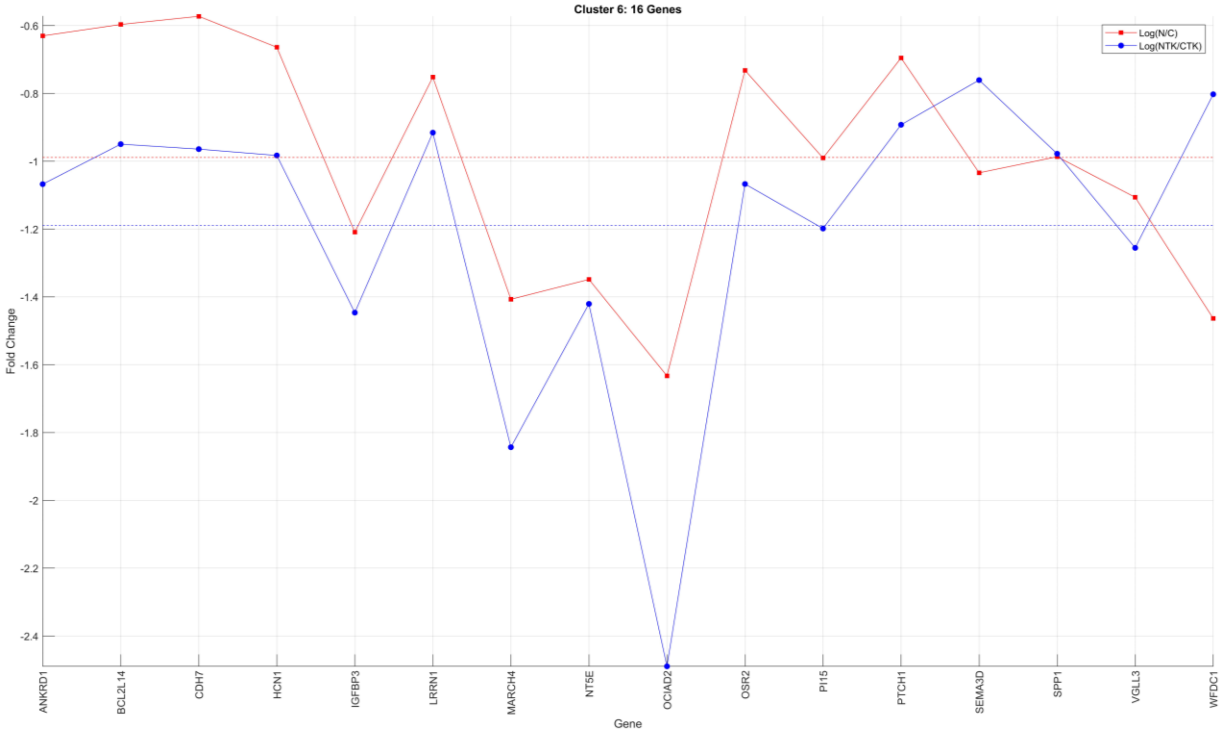

Fig. S2. Changes in activities of genes.

(A) Histogram shows logarithmic fold change in intersecting groups A-B-D and A-B-C-D (see also Fig. 1A,B); inset is the empirical *cumulative distribution function* of the two graphs. No significant differences between NCC/NPC and NCC^{TK-}/NPC^{TK-} were observed in these groups. (**B**) Relationship between fold changes in the activities of the 475 genes common to groups A, C, and D during NPC$\rightarrow$NCC transition versus those during $NPC^{TK-}\rightarrow NCC^{TK-}$ transition (i.e., when nFGFR1 function was reduced). Slope illustrates difference between NCC^{TK-}/NPC^{TK-} and NCC/NPC. Cluster 5 [Log(FC) of ~1] (**C**), cluster 2 [Log(FC) of ~2.4] (**D**), and cluster 1 [Log(FC) = 2] (**E**) from Fig. 1D: most genes have higher Log(FC) for NCC^{TK-}/NPC^{TK-} than for NCC/NPC. Cluster 3 [Log(FC) = -0.3] (**F**) and cluster 6 [log(FC) = -1] (**G**) from Fig. 1D: more genes have lower Log(FC) for NCC^{TK-}/NPC^{TK-} than for NCC/NPC; an exception is *C7ORF43* in cluster 3 (FC changes from negative FC in NCC/NPC to a positive change in NCC^{TK-}/NPC^{TK-}.

A.

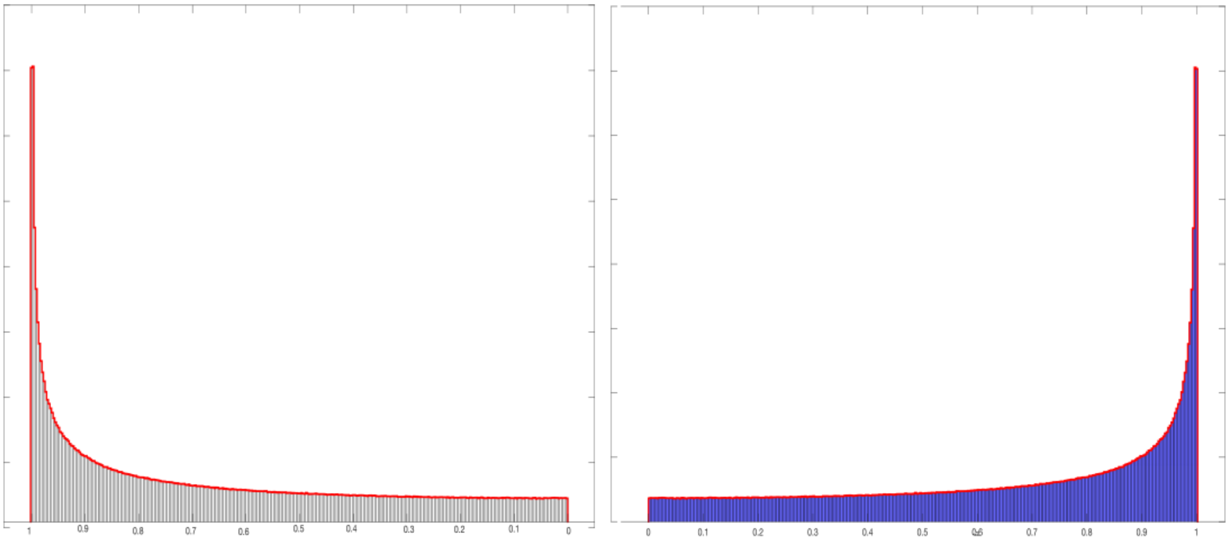

B.

Positive correlations *r* 0 to +1.00							Negative correlations *r* -1.0 to 0						
	α	β	mean	std dev	**threshold 95%**			α	β	mean	std dev	**threshold 95%**	
C	0.95153	0.4946	0.658	0.3033	**0.9613**		C	0.9492	0.4983	-0.6558	0.3037	**-0.9595**	
CTK	0.9577	0.49424	0.6596	0.3026	**0.9622**		CTK	0.9364	0.5039	-0.6502	0.3053	**-0.9555**	
N	0.96477	0.48091	0.6673	0.3013	**0.9686**		N	0.9625	0.4824	-0.6661	0.3016	**-0.9677**	
NTK	0.95701	0.48484	0.6637	0.3023	**0.9661**		NTK	0.9426	0.5065	-0.6505	0.3047	**-0.9552**	
NLS	0.94657	0.5026	0.6532	0.3041	**0.9573**		NLS	0.9218	0.5107	-0.6435	0.3071	**-0.9506**	

C.

Positive correlations *r* 0 to +1.00							Negative correlations *r* -1.0 to 0						
	α	β	mean	std dev	**threshold 95%**			α	β	mean	std dev	**threshold 95%**	
Control	1.00468	0.94037	0.5165	0.2912	**0.8077**		Control	1.0047	0.9404	-0.5165	0.2912	**-0.8077**	
Schiz	1.02248	0.80546	0.5594	0.2952	**0.8546**		Schiz	1.0225	0.8055	-0.5594	0.2952	**-0.8546**	

Fig. S3. Critical *r* thresholds.

Identification of critical *r* threshold values for NPCs and NCCs (*n* = 16,137 genes) [(*7*)and for NCCs cultured from induced pluripotent stem cells derived from patients with schizophrenia (Schiz) and from controls (*n* = 15,279 genes)[10]. (**A**) To identify 95%

critical (threshold) values of each *r* from the Pearson frequency distribution, the positive (>0 to +1) and negative (<0 to -1) *r* frequency data were fit to s β model (example shown); see "*Gene–gene coordination represented via correlations*" in the supplemental text for details. (**B**,**C**) Mean (±SD) critical threshold values were identified for the positive and negative correlations fit to β model.

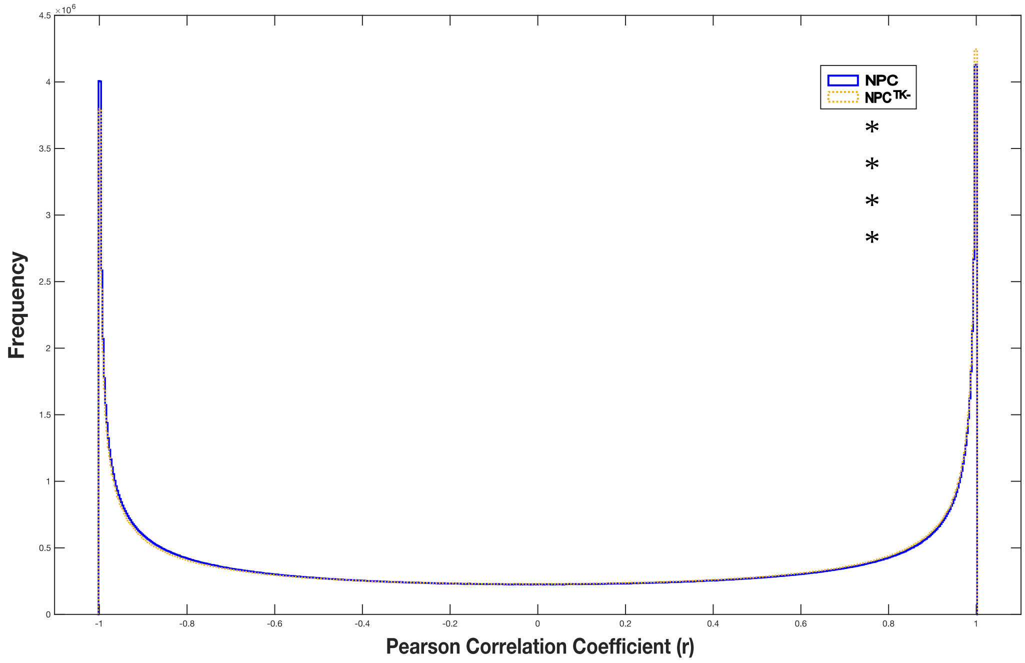

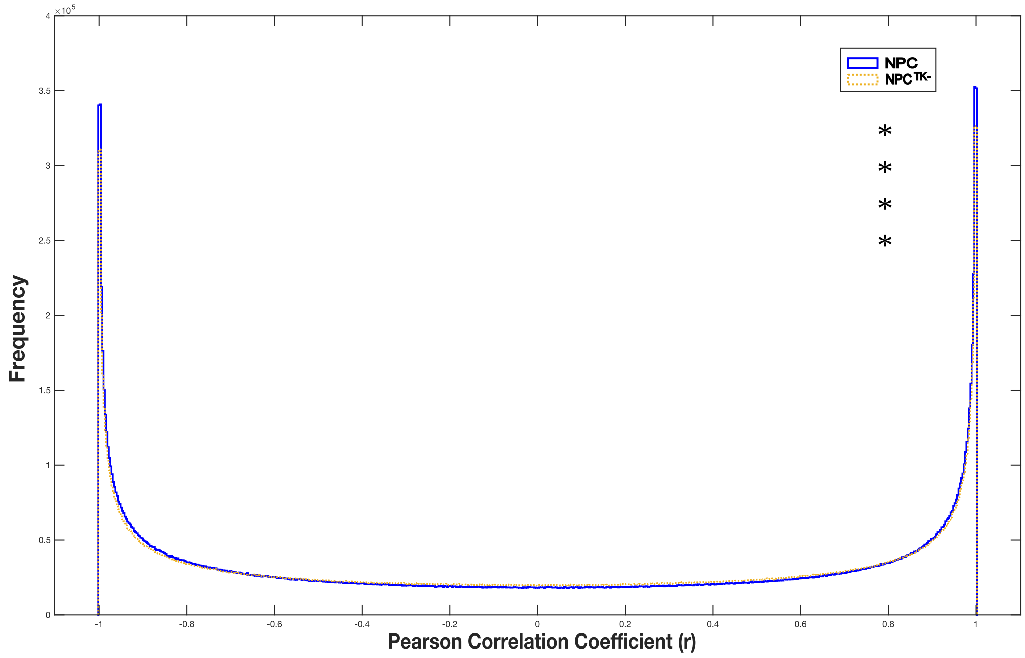
NPC
NPC TK-
*
*
*
*
Frequency
Pearson Correlation Coefficient (r)

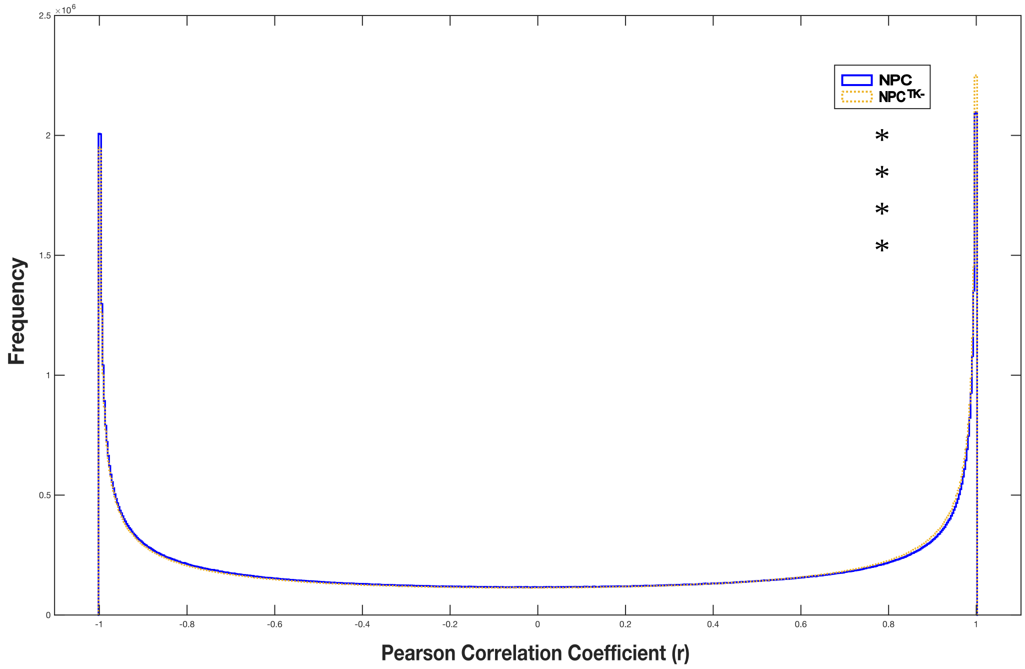
NPC
NPC TK-
*
*
*
*
Frequency
Pearson Correlation Coefficient (r)

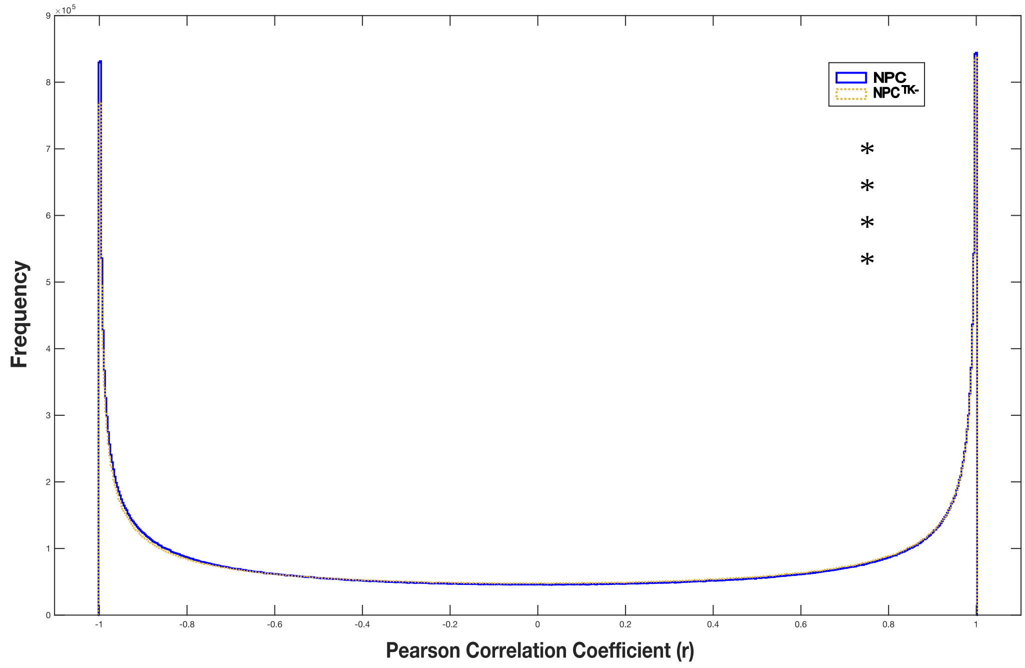

Fig. S4. Frequency distributions of Pearson correlations for genes in NPCs and NPC^{TK-}s (entropy values are shown in Fig. 3F). (**A**) All 16,137 genes expressed; (**B**) 4,646 Reg genes (average activities changed during NPC→NCC transition); (**C**) 11,491 non-Reg genes (no change in average activity during transition); (**D**) Cross-correlation of Reg and nonReg genes.. χ^2 tests ****P < 0.0001. Entropy changes associated with the correlation frequency distributions are listed in Table S1.

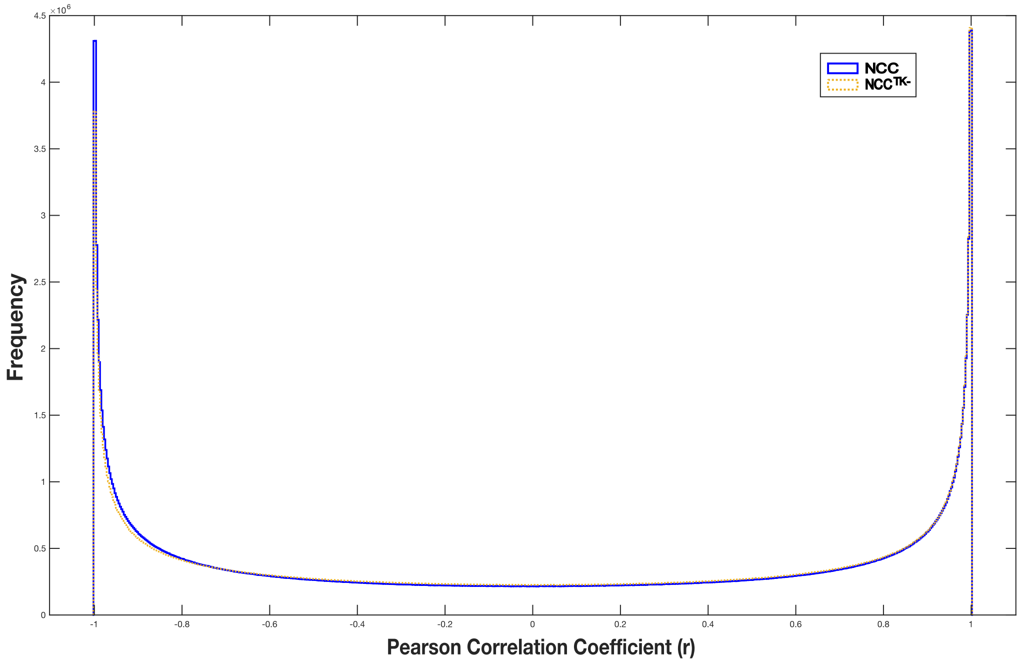
NCC
NCC^{TK-}
Frequency
Pearson Correlation Coefficient (r)
×10^6

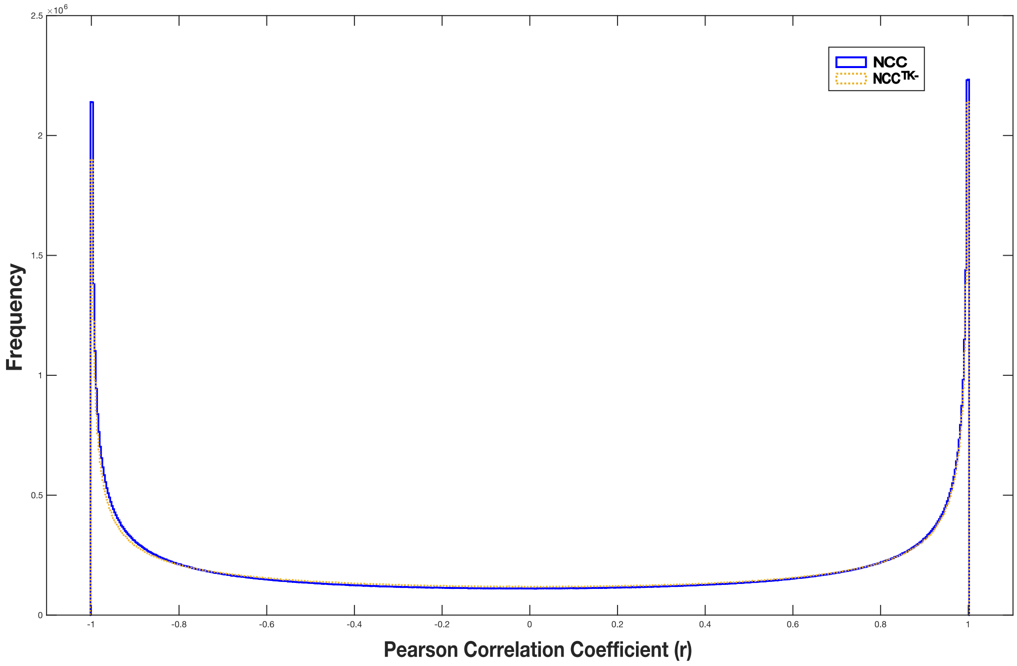
Frequency
Pearson Correlation Coefficient (r)
NCC
NCC^TK-

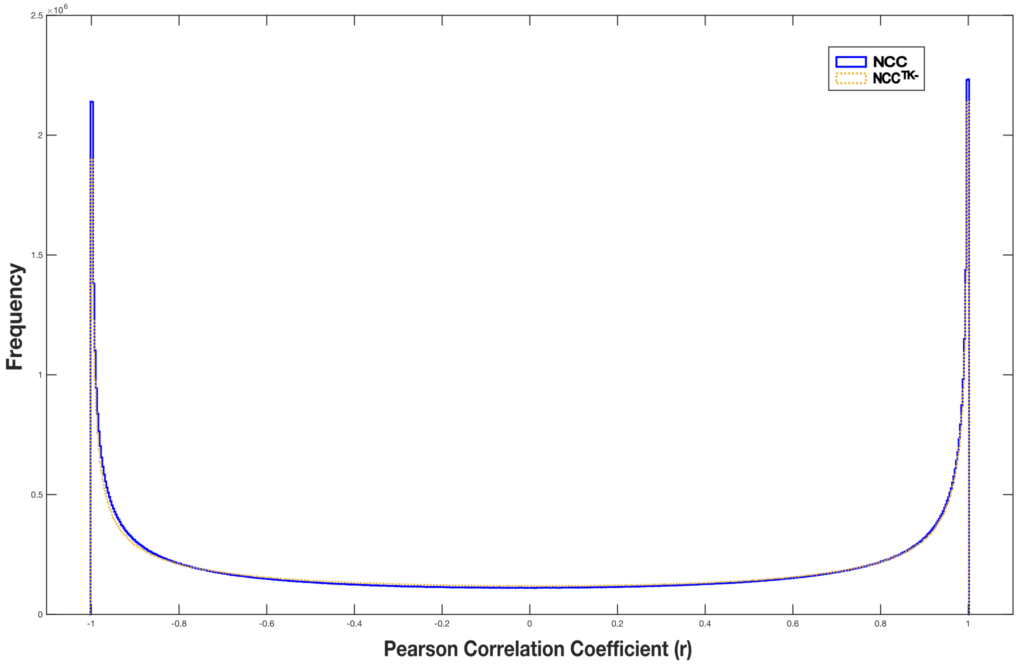
Frequency
Pearson Correlation Coefficient (r)
NCC
NCC^TK-

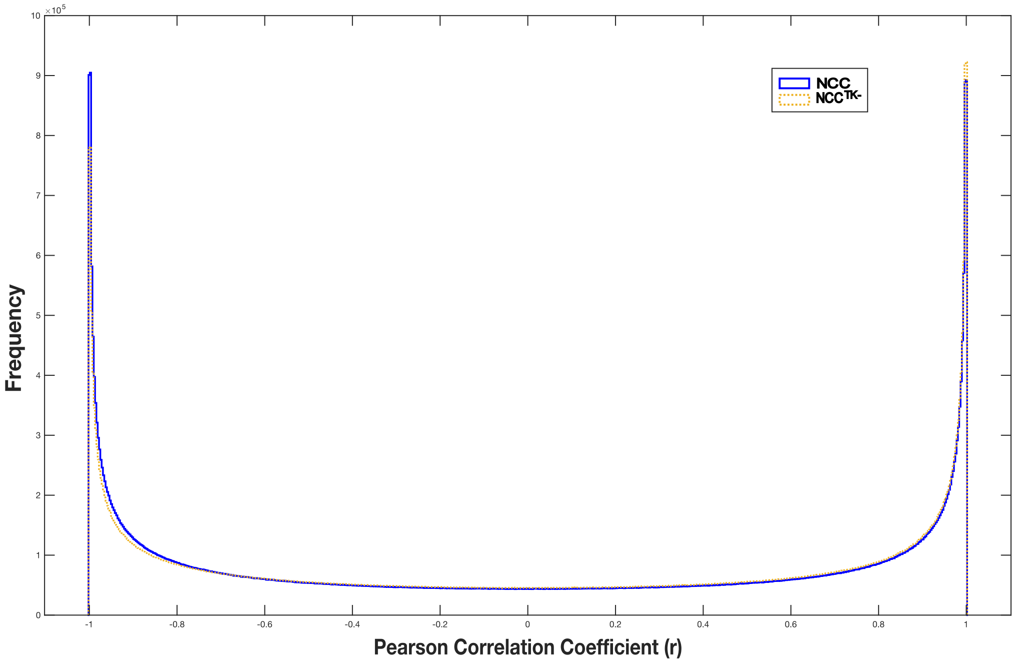

\

Fig S5. Frequency distributions of Pearson correlations for genes in NCCs and NCC^{TK-}s -

(**A**) All 16,137 genes expressed; (**B**) 4,646 Reg genes (average activities changed during NPC→NCC transition); (**C**) 11,491 non-Reg genes (no change in average activity during transition); (**D**) Cross-correlation of Reg and nonReg genes. χ^2 tests **** $P < 0.0001$. Entropy changes associated with the correlation frequency distributions are listed in Table S1.

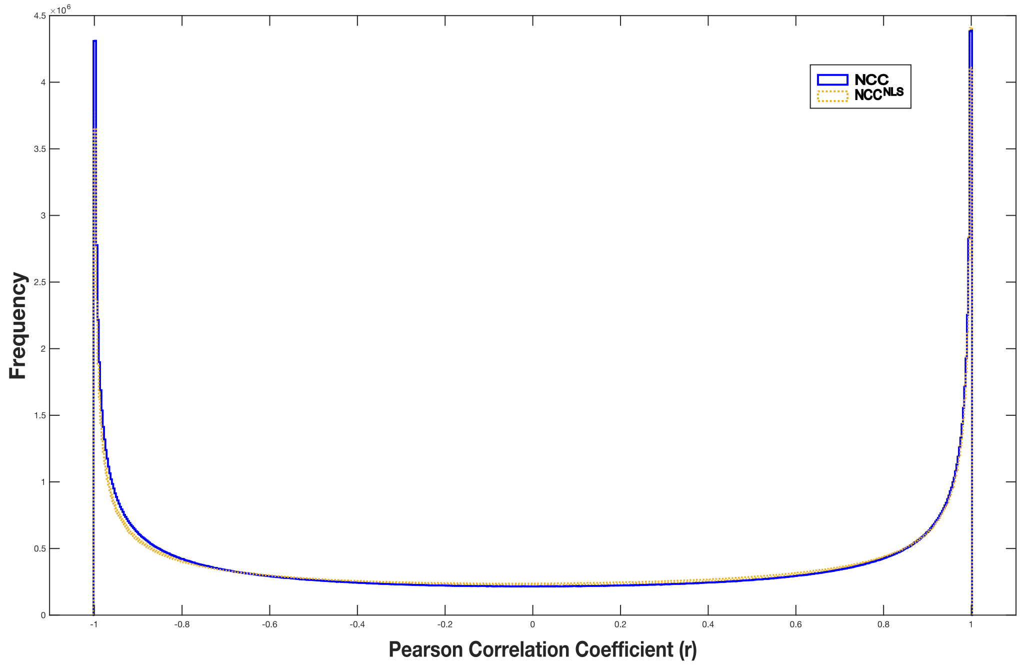
NCC
NCC^NLS
Frequency
Pearson Correlation Coefficient (r)

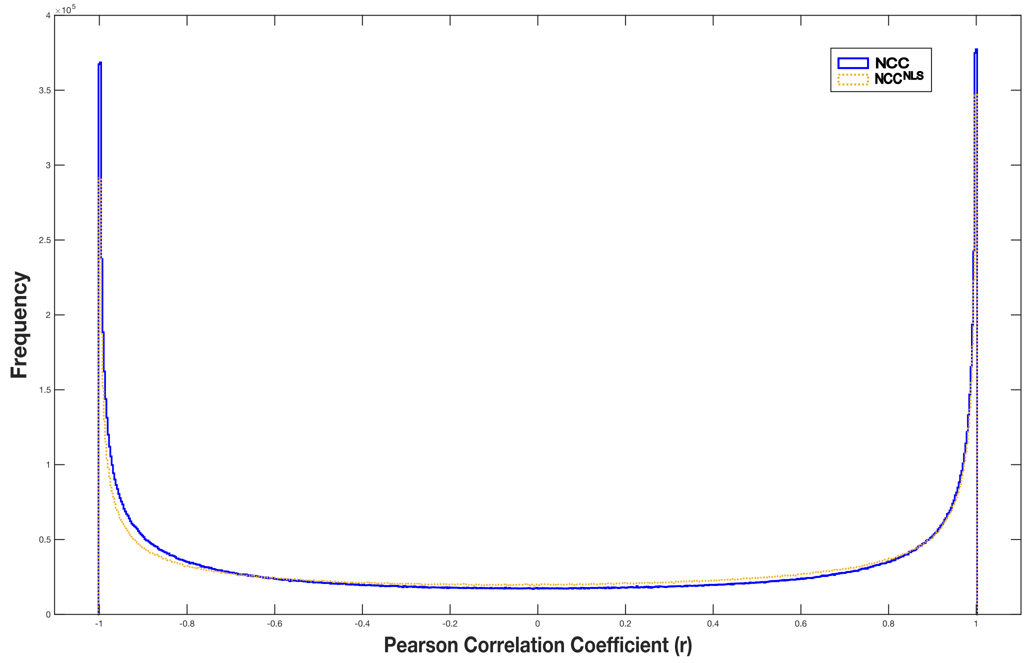
NCC
NCC^NLS
Frequency
Pearson Correlation Coefficient (r)

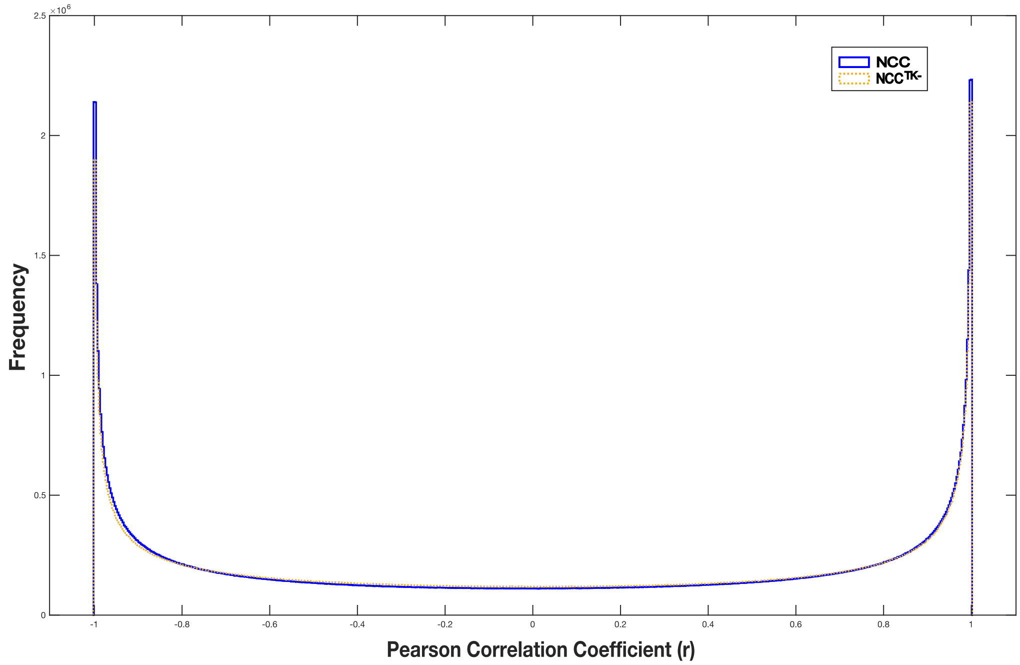

NCC
NCC^TK-
Frequency
Pearson Correlation Coefficient (r)

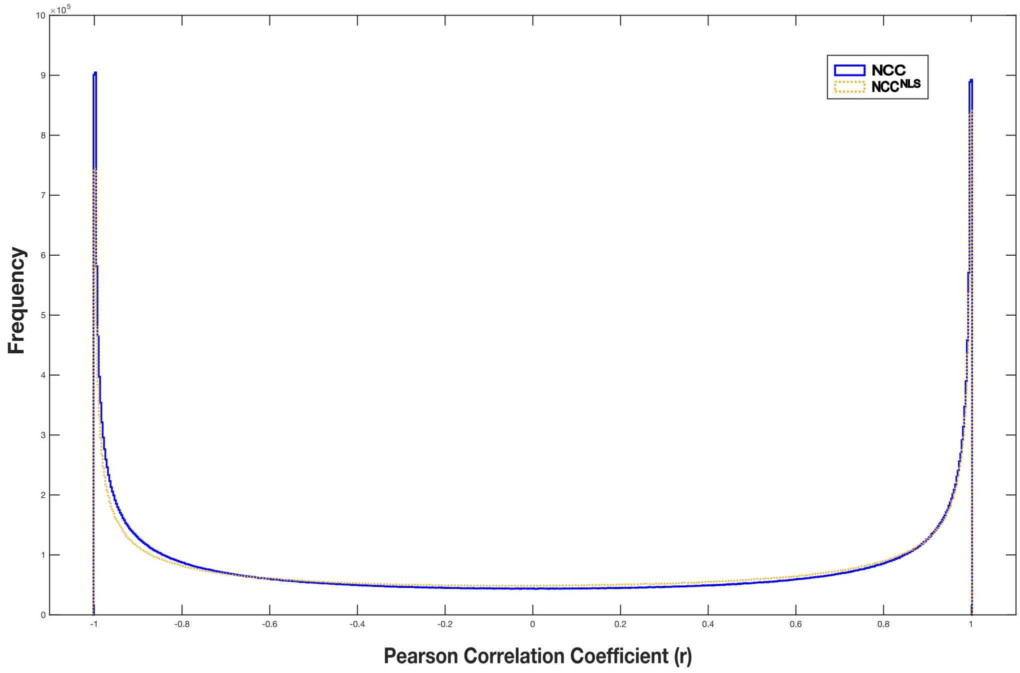

NCC
NCC^NLS
Frequency
Pearson Correlation Coefficient (r)

Fig. S6. Frequency distributions of Pearson correlations for genes in NCCs and NCC^{NLS}s.

(**A**) All 16,137 genes expressed; (**B**) 4,646 Reg genes (average activities changed during NPC→NCC transition); (**C**) 11,491 non-Reg genes (no change in average activity during transition); (**D**) Cross-correlation of Reg and nonReg genes. χ^2 tests ****$P < 0.0001$. Entropy changes associated with the correlation frequency distributions are listed in Table S1.

A

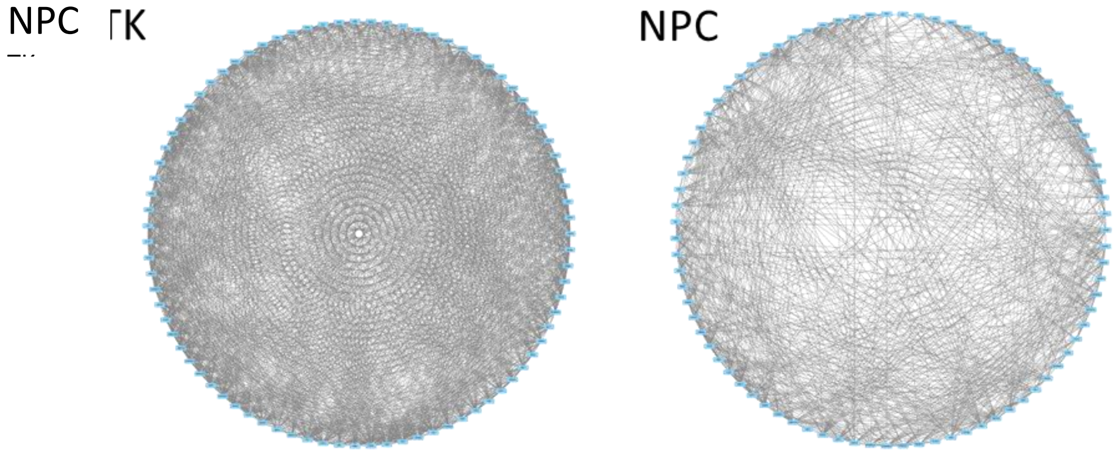

B

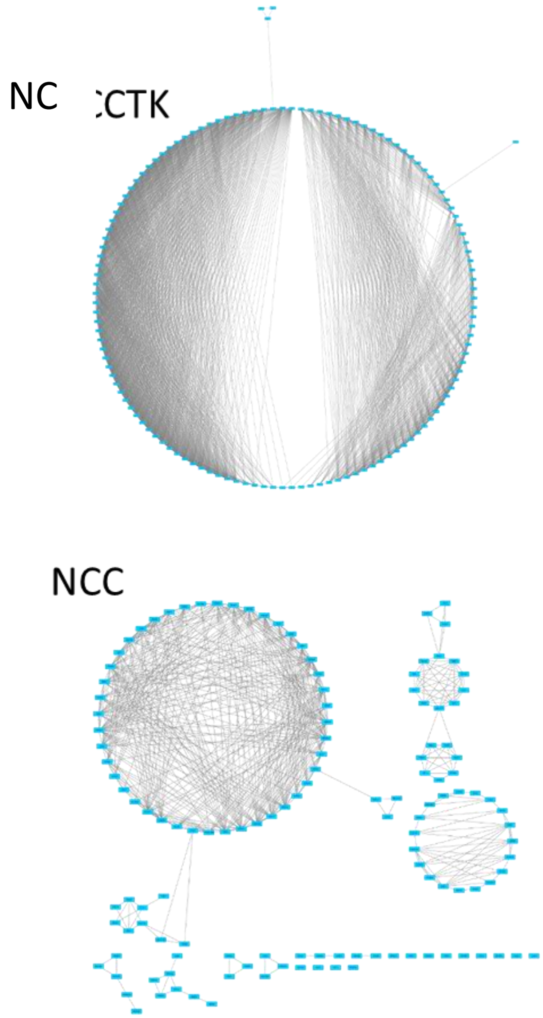

C

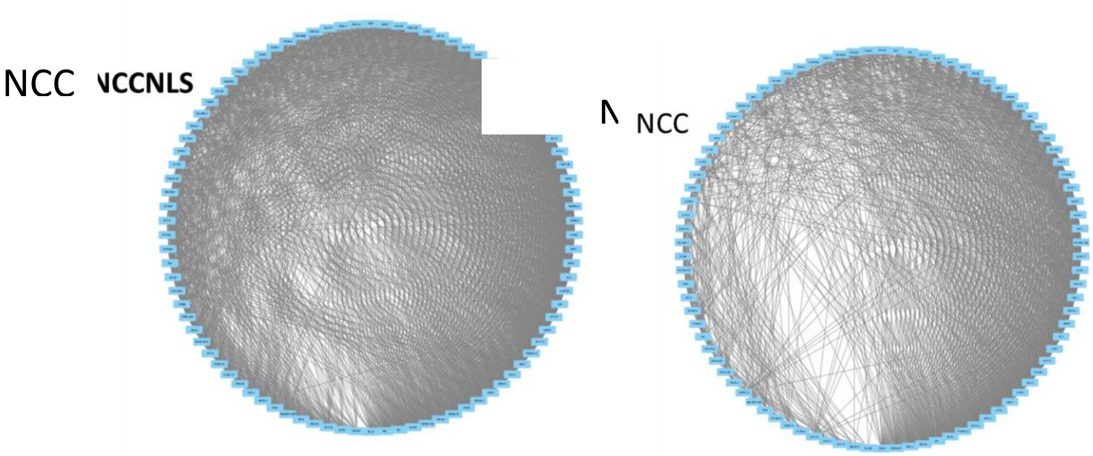

D

Network characteristics:	$\mathbf{NPC^{TK-}_{ref}}$	NPC	$\mathbf{NCC^{TK-}_{ref}}$	NCC	$\mathbf{NCC^{NLS}_{ref}}$	NCC
No of nodes	83	83	131	131	90	90
No of edges	1733	954	2118	508	2150	1439
Average No of neighbors	42	22.99	32.33	7.76	47.78	31.98
Network diameter	4	6	6	5	4	5
Characteritic path length	1.537	1.984	1.564	1.76	1.487	1.782
Clustering coeficient	0.382	0.311	0.409	0.292	0.382	0.341
Network density	0.255	0.14	0.124	0.03	0.268	0.18

Fig. S7. Correlations of the Reg nervous system development (NSD) genes.

Circular network analysis of genes with Pearson coefficients (*r* values) of >+0.99 (strongly correlated). Reg genes are those whose average activity was altered by FGFR1(SP-/NLS)(TK-). (**A**) Gene activity network (GAN) formed by 83 strongly correlated NPC^{TK-} genes compared to the same genes in NPCs. (**B**) GANs formed by 131 NSD genes (*r* > 0.99) in NCC^{TK-}s compared to correlations of the same genes in NCCs. (**C**) GANs formed by 90 NSD genes in NCC^{NLS}s compared to those in NCCs. The genes represent Reg genes whose average activity was altered by FGFR1(SP-/NLS). (**D**)

Summary statistics: circular network characteristics are compared to a reference network **(ref)** condition for a given group of genes.

	r > 0.96		r < - 0.96		r -1 to +1	
	No. of Pairs	r_{avg} ±SEM	No. of Pairs	r_{avg} ±SEM	r_{avg} ±SEM	Entropy
NPC	2270	0.9903 ±0.0001	2215	-0.9904 ±0.0001	-0.0005 ±0.004	S=4.812
NPC TK-	2184	0.9897 ±0.0001 *	2228	-0.9901 ±0.0001	0.0019 ±0.0043 ***	S=4.845 $\Delta S_{NPC-NPCTK-}$ +0.03
NCC	3074	0.9904 ±0.0001	2762	-0.9904 ±0.0001	0.0239 ±0.0046	S=4.7762 $\Delta S_{NPC-NCC}$ -0.036
NCC TK-	1951	0.9896 ±0.0002 **	1946	-0.9896 ±0.0002 **	-0.0025 ±0.0042 ***	S=4.8621 $\Delta S_{NCCTK- - NCC}$ +0.085
NCC NLS	2342	0.9903 ±0.0001	2153	-0.9899 ±0.0001	0.0218 ±0.0043	S=4.905 $\Delta S_{NCCNLS - NCC}$ +0.138

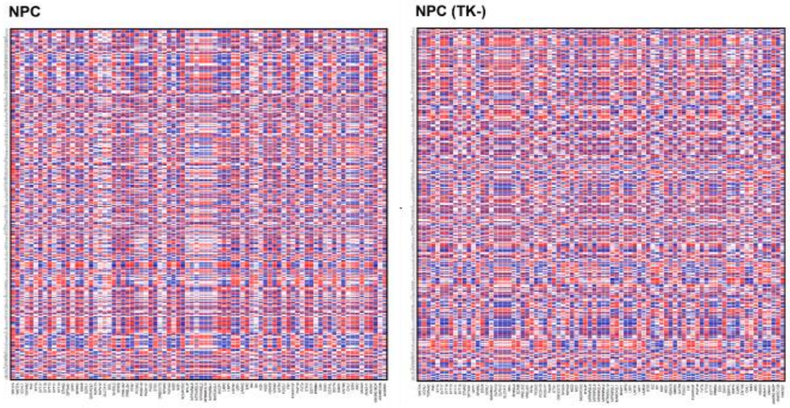

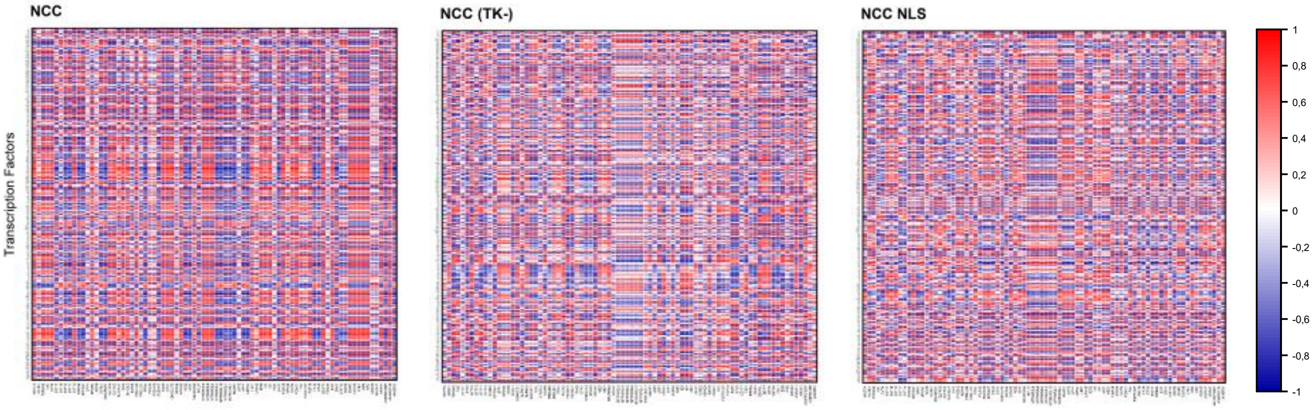

Fig. S8. Cross-correlation of transcription factor genes with nontranscription factor genes.

The genes pairs with positive (red) or negative (blue) correlations were identified within the 4,646 Reg genes. Heat maps of correlations and quantitative analysis of correlations are shown. *$P < 0.05$, **$P < 0.01$, ***$P < 0.001$; S, entropy; ΔS, entropy change.

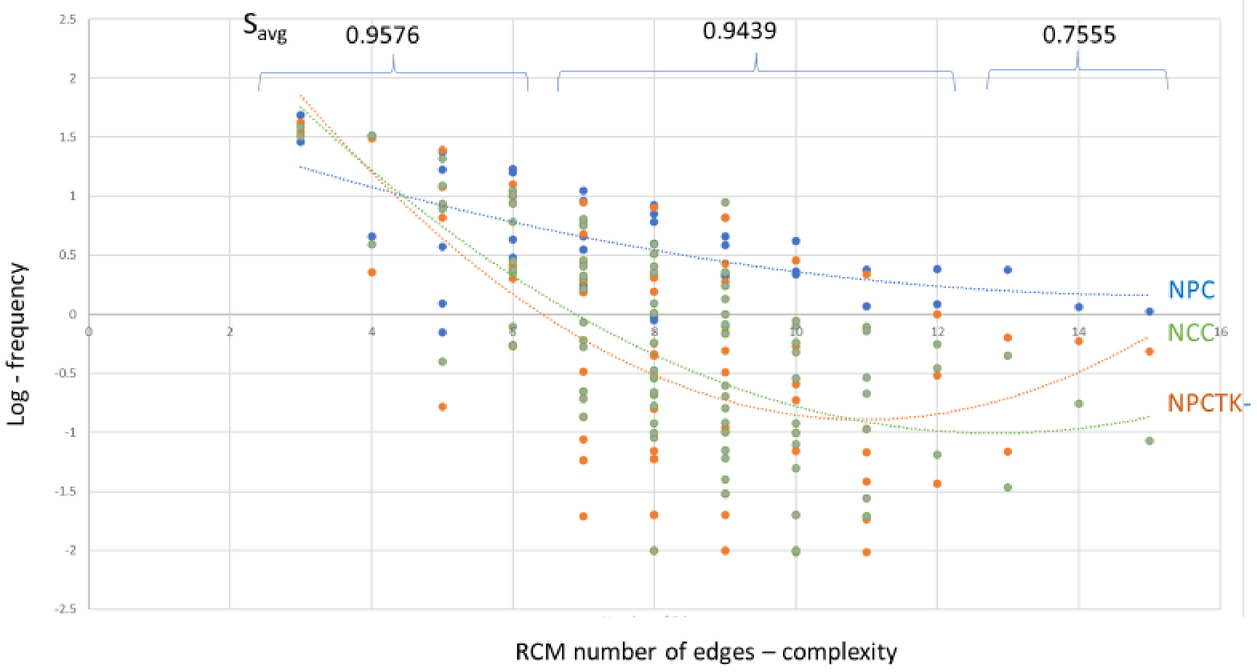
S_avg
0.9576
0.9439
0.7555
NPC
NCC
NPCTK-
Log - frequency
RCM number of edges – complexity

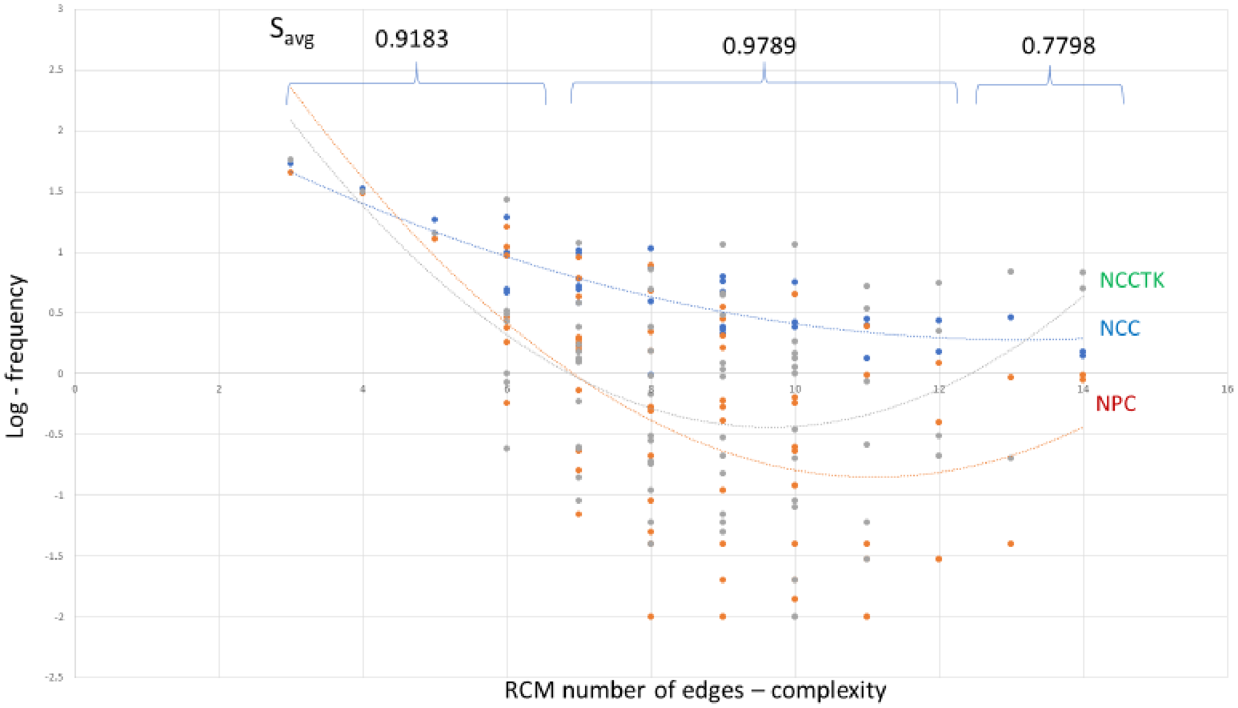
S_avg
0.9183
0.9789
0.7798
NCCTK
NCC
NPC
Log - frequency
RCM number of edges – complexity

Fig. S9. Three-six-node recurring coordination modules (RCMs) overrepresented in nervous system development (NSD) gene activity networks (GANs).

Shown are the relationships between complexity (measured by number of edges), entropy, and the frequency of RCMs. (**A**) RCMs of the NPC 83-gene NSD GAN (Fig. 3A and 4A) compared to GANs of the same genes in NPC^{TK-} and NCC GANs. The average entropy (S_{avg}) values were calculated for low complexity (3–6 edges), medium complexity (7–12 edges), and high complexity (13–15 edges) modules. *Commentary: Frequency of the NPC GAN RCM declined between the simple 3-edge RCMs and the most complex 15-edge RCMs. The same GAN in NCCs had reduced frequency of the medium-complexity/high-entropy RCMs. In NPC^{TK-}s, frequencies of the medium-complexity/high-entropy RCMs as well as of the high-complexity/low-entropy RCMs decreased. Thus, endogenous nFGFR1 promotes formation of both the medium-complexity/high-entropy and high-complexity/low-entropy RCMs in the NPC GAN.* (

B) RCMs of the 131-gene NSD GAN (Fig. 3B and 4B) compared to GANs of the same genes in NCC^{TK-}s and NPCs. The S_{avg} values were calculated as described above. *Commentary: Frequency of the NCC RCMs declined between the simple 3-edge RCMs and the most complex 15-edge RCMs. The same GAN in NPCs had reduced frequency of the medium-complexity/high-entropy RCMs. In NPC^{TK-}s, frequencies of the medium-complexity/high-entropy RCMs decreased; however, the high-complexity/low-entropy RCMs were similar as in NCCs. Thus, endogenous nFGFR1 promotes formation of the medium-complexity/high-entropy RCMs in the NCC GAN.*

A

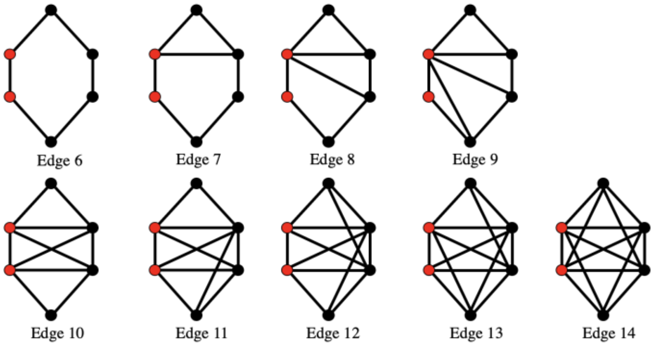

B

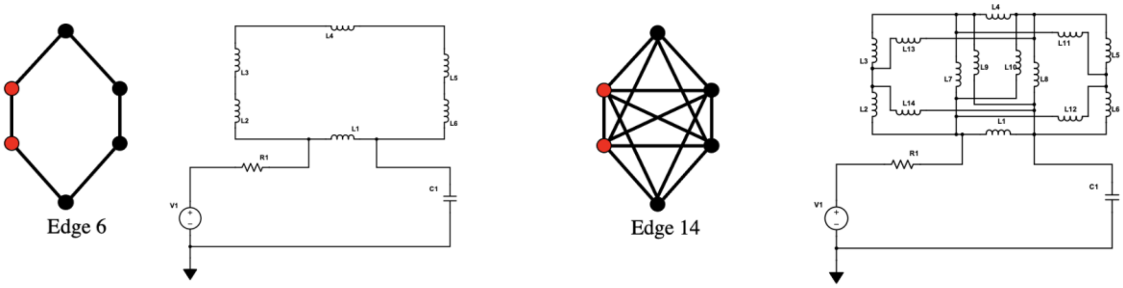

C

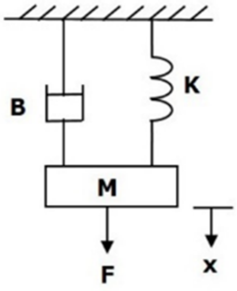

F = force
M = mass
K = spring constant
B = friction coefficient
X = displacement

**Mechanical System**	**Electrical System**
**Force(F)**	Voltage(V)
**Mass(M)**	Inductance(L)
**Frictional Coefficient(B)**	Resistance(R)
**Spring Constant(K)**	Reciprocal of Capacitance (1/c)
**Displacement(x)**	Charge(q)
**Velocity(v)**	Current(i)

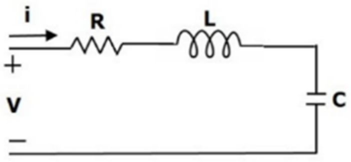

R = resistor
L = inductor
C = Capacitor
I = current
V = voltage

D

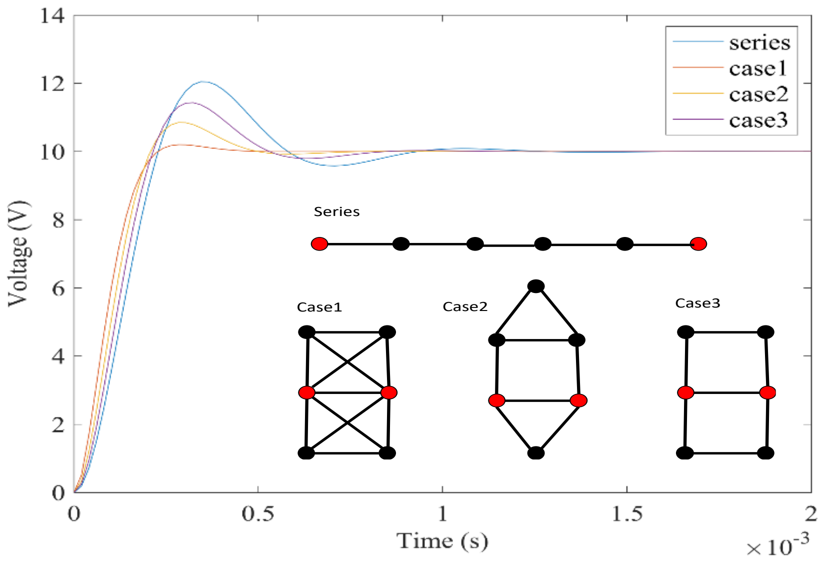

Fig. S10. Modeling RCMs as electrical R-C-L networks

(**A**) Different modules that are possible with six nodes. The red nodes are the active nodes where the signal is being transmitted from one node to another. The rest of the network influences the net inductance between these two nodes, effectively controlling signal dampening. (**B**) R-L-C equivalent circuit models. (**C**) Force transfer system equivalent to R-L-C circuit: R, L, and C correspond to friction, mass/moment of intertia, and inverse of the spring constant, respectively.

(**D**) The step response of R-L-C circuits showing the effects of topology (connectivity) on circuit response to a stable change in the input energy.

Commentary: In an R-L-C circuit supplied with energy, R (resistor, Ohms) causes a loss of energy and restricts the signal flow, which in the genome reflects the innate resistance of gene promoters and epigenomic structures to modifications. L (inductor, Henry) is the phaser element; it initially blocks the alternating current (changing energy), and then it sends it through with an added phase/delay added; in the genome, it reflects the time required to evoke the change in activity of the next node (gene) in the network, causing

delay (and a change of phase). C (capacitor, Farad) is also a phasor element; it blocks the direct current (continuous energy) but allows alternating current (time varying) through while also storing energy; in the genome, it reflects mechanical energy stored in looped or supercoiled DNA as well as subthreshold accumulation of an effector. Oscillations are produced by the combination of L and C, and the frequency and sensitivity to the oscillations is determined by the values of L and C. The value of R changes the slope because it restricts energy; the oscillations dampen because it consumes the energy and returns the system to a stable equilibrium state. In fig. S10A, B we observe that for inductor (L) circuits in a more connected parallel arrangement, the noise-dampening function is increased in such a manner that demonstrates a higher dampening function of the signals.

A

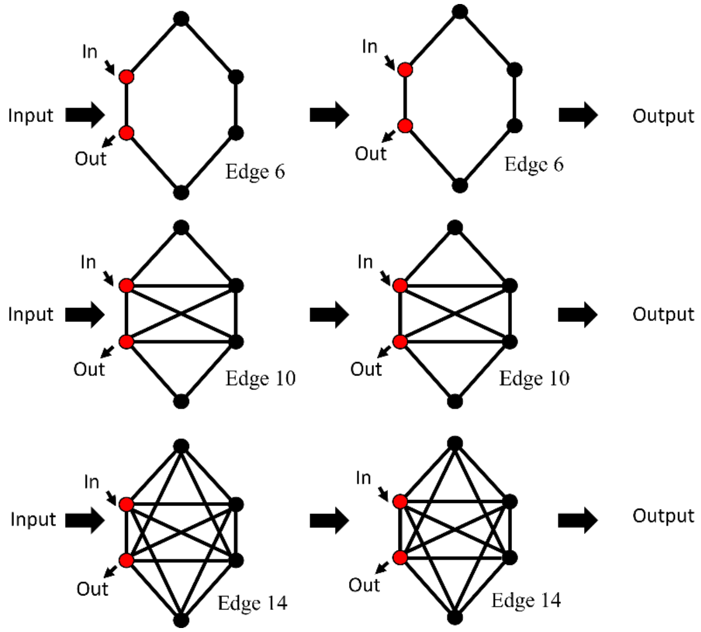

In
Out
Input
Output
Edge 6
Edge 10
Edge 14

B

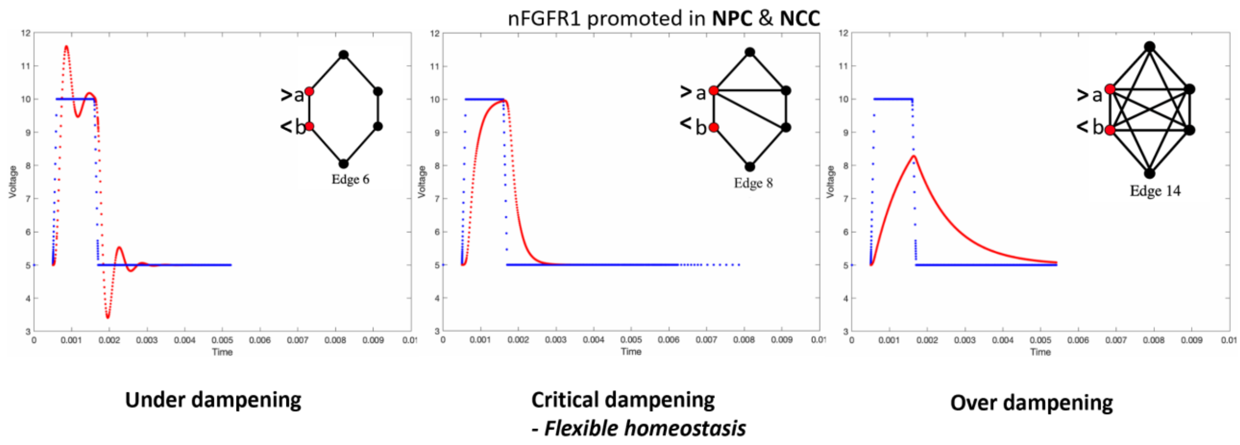

nFGFR1 promoted in **NPC** & **NCC**
Voltage
Time
>a
<b
Edge 6
Edge 8
Edge 14
Under dampening
Critical dampening
- *Flexible homeostasis*
Over dampening

C

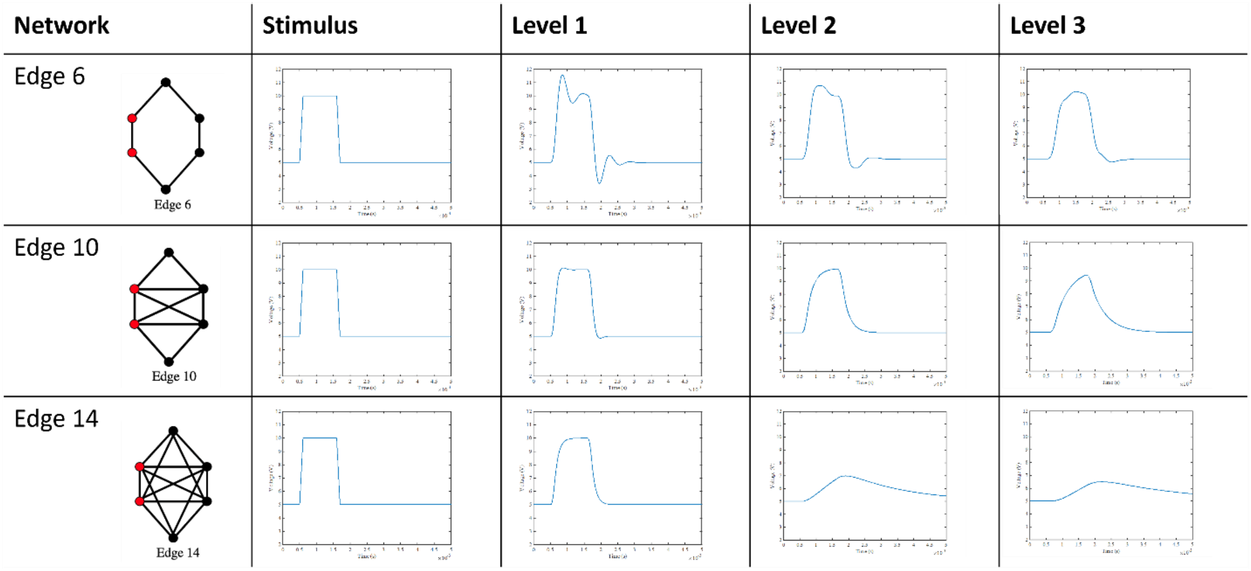

Fig. S11. Signal (information) transmission between GAN modules.

(**A, B**) Two-level cascading of 6-, 8-, and 14-edge motifs: effect of underdampening, critical dampening, and overdampening on information transfer. Panels illustrate the effect of motif complexity on signal transmission to genes outside the motif: nodes *a* and *b* are the motif's receiving and communicating nodes, respectively; responses would be measured by a node outside the motif. (**C**) Further amplification of the dampening and information transfer in level 3 cascading. *Commentary: Building on these fundamental modules of 6-node circuits, the effect of cascading such a system can be demonstrated. The output of the first module was fed to the input of the second module (as shown in panel A). The output after the second block in response to the stimulus is shown in panel B). For the cascaded 6-edge module, oscillations are observed in the output of the cascade. Eight edges provide a critically damped case: there is effective information transfer without corruption by added oscillations. For 14-edge motifs, the overdampening effect of the cascading restricts the flow of information.*

Supplemental Table S1. Summary of the entropy values (S) and entropy changes (ΔS) in *r* frequency histograms.

histograms - figures 3A-D, S4-S6		All expressed genes		Reg genes		Reg - nonReg genes		nonReg		NSD genes	(Fig.3A-D)
		S	ΔS	S	ΔS	S	ΔS	S	ΔS	S	ΔS
NPC		4.835		4.092		4.7889		4.8609		4.905	
NPCTK-	ΔS=NPCTK- - NPC	4.928	(+) 0.093	4.167	(+)0.075	4.882	(+)0.093	4.954	(+)0.093	4.85	(-)0.055
NCC	ΔS=NCC - NPC	4.804	(-) 0.031	4.1	(+)0.009	4.739	(-)0.050	4.8412	(-)0.049	5	(+)0.095
NCCTK-	ΔS=NCC^{TK-} - NCC	4.904	(+) 0.100	4.157	(+)0.057	4.883	(+)0.144	4.8914	(+)0.050	4.4352	(-)0.565
NCCNLS	ΔS=NCC^{NLS} - NCC	4.965	(+) 0.161	4.272	(+)0.172	4.945	(+)0.206	4.925	(+)0.084	5.0825	(+)0.083

histograms - figure 3E		All expressed genes		dysReg genes		non-dysReg		dysReg - non-dysReg	
		S	ΔS	S	ΔS		ΔS		ΔS
Control NCC		4.979		4.951		4.98		4.9705	
Schiz NCC	ΔS=Schz - Control	4.916	(-)0.063	4.777	(-)0.174	4.9255	(-)0.055	4.8662	(-)0.105

Supplemental Table S2. Table shows r_{avg} $\underline{+}$SEM and number of pairs (n) for the same group of genes which in the reference condition **(bold)** had r > threshold confidence value of >95% (A) Correlation of all genes expressed, (B) 4646 Reg genes, (C) cross-corrleated 4,646 Reg genes with 11,136 nReg genes.

conditions	Positive corr.				Negative corr.			
compared	Number of Pairs	Mean	SEM	p (ANOVA)	Number of Pairs	Mean	SEM	p (ANOVA)
NPC^{TK-}	25607112	0.167363682	2.77231E-08	<0.0001	25468915	-0.110444976	2.79032E-08	<0.0001
NCC	25607094	0.118391145	2.84627E-08	<0.0001	25468915	-0.108629728	2.86412E-08	<0.0001
NCC^{NLS}	25607112	0.166410153	2.75583E-08	<0.0001	25468915	-0.104391991	2.7837E-08	<0.0001
NCC^{TK-}	25607112	0.121964006	2.79943E-08	<0.0001	25468915	-0.055821492	2.83318E-08	<0.0001
**NPC > 0.9613**	25607112	0.987133884	4.50209E-10		25468915	-0.986512918	4.73863E-10	
NPC	24410143	0.127639141	2.94163E-08	<0.0001	24374365	-0.094047752	2.93603E-08	<0.0001
NPC^{TK-}	24410143	0.142809655	2.92839E-08	<0.0001	24374365	-0.117140801	2.94815E-08	<0.0001
NCC^{NLS}	24410143	0.270045378	2.7881E-08	<0.0001	24374365	-0.210394125	2.81407E-08	<0.0001
NCC^{TK-}	24410143	0.147866774	2.89871E-08	<0.0001	24374365	-0.098506973	2.92918E-08	<0.0001
**NCC > 0.9686**	24410143	0.989594906	3.82801E-10		24374365	-0.989282407	3.94443E-10	
NPC^{TK-}	2176132	0.013743481	3.2767E-07	<0.0001	2162342	0.008047537	3.30E-07	<0.0001
NCC	2176132	0.070463895	3.39756E-07	<0.0001	2162342	-0.055631461	3.42E-07	<0.0001
NCC^{NLS}	2176132	0.141490615	3.2481E-07	<0.0001	2162342	-0.050652362	3.29E-07	<0.0001
NCC^{TK-}	2176132	0.135845809	3.36897E-07	<0.0001	2162342	-0.015949404	3.43E-07	<0.0001
**NPC > 0.9613**	2176132	0.987163402	5.2976E-09		2162342	-0.986524513	5.58E-09	
NPC	2090844	0.06745943	3.49E-07	<0.0001	2077562	0.004500266	3.43E-07	<0.0001
NPC^{TK-}	2090844	0.015700697	3.41E-07	<0.0001	2077562	-0.066779266	3.49E-07	<0.0001
NCC^{NLS}	2090844	0.28986333	3.21E-07	<0.0001	2077562	-0.203926702	3.29E-07	<0.0001
NCC^{TK-}	2090844	0.172021203	3.45E-07	<0.0001	2077562	-0.066454023	3.53E-07	<0.0001
**NCC > 0.9686**	2090844	0.989619416	4.46E-09		2077562	-0.989299394	4.62E-09	
NPC^{TK-}	1048575	0.151850105	6.75E-07	<0.0001	5275181	-0.115578277	1.34E-07	<0.0001
NCC	1048575	0.084971707	6.98E-07	<0.0001	5275181	-0.096261802	1.39E-07	<0.0001
NCC^{NLS}	1048575	0.14736759	6.73E-07	<0.0001	5275181	-0.088811366	1.35E-07	<0.0001
NCC^{TK-}	1048575	0.111589972	6.91E-07	<0.0001	5275181	-0.047270998	1.37E-07	<0.0001
**NPC > 0.9613**	1048575	0.987158698	1.10E-08		5275181	-0.986521941	2.29E-09	
NPC	4950819	0.102968928	1.46E-07	<0.0001	5104920	-0.091090808	1.40E-07	<0.0001
NPC^{TK-}	4950819	0.126607748	1.44E-07	<0.0001	5104920	-0.099450722	1.41E-07	<0.0001
NCC^{NLS}	4950819	0.274895722	1.37E-07	<0.0001	5104920	-0.21064046	1.34E-07	<0.0001
NCC^{TK-}	4950819	0.144933896	1.43E-07	<0.0001	5104920	-0.090125153	1.40E-07	<0.0001
**NCC > 0.9686**	4950819	0.989598942	1.89E-09		5104920	-0.989287019	1.88E-09	

Supplemental Table S3A. NSD genes (83 genes from clustered NPC network in Fig. 5A and the circular GAN in Fig. 4A) whose average activity was altered in NPC^{TK}; cluster assignment and number of connections with other genes are listed. The gene functions were assigned based on g:Profiler hsapiens and Reactome.

Gene No network	Edges NPC	Edges NPCTK	NPC Cluster I	Gene name	Function/Pathway
63	37	5	Cluster I	ANKRD18DP	Ankyrin repeat domain 1 pleyotropic protein, transcription, development
33	46	2	Cluster I	LRFN1	Protein-protein interactions at synapses
36	45	3	Cluster I	DDIT4	DNA damage induced protein, TP53 signaling
42	43	5	Cluster I	FGF19	Signaling by FGFR4, MAPK signaling
47	42	4	Cluster I	EID1	P300 binding differentiation inhibitor
60	38	3	Cluster I	PRELID2	PRELI domain containing 2 protein nvolved in embryonic development., phospholipid transfer.
66	35	0	Cluster I	OTX1	Neurodevelopmental TF
68	34	3	Cluster I	EGR2	EGR2 and SOX10 myelination, NGF/NRTKs sigaing, HOX genes activation
69	34	1	Cluster I	DDIT4L	DNA damage induced protein,
76	31	1	Cluster I	MTRNR2L10	Humanin-like 10 neuroprotective and antiapoptotic;
77	31	4	Cluster I	MTRNR2L2	Humanin-like 2 neuroprotective and antiapoptotic; mitochondrial
79	31	1	Cluster I	MTRNR2L1	Humanin-like 1 neuroprotective and antiapoptotic; mitochondrial
54	40	3	Cluster II	LEF1	RUNX3 regulates WNT signaling, gastrulation, beta-catenin regulation
35	45	2	Cluster II	ANXA1	Signaling by Interleukins, GPCR signaling
20	49	1	Cluster II	ANXA11	Anexin 11 calcium binding phospholipid dependent, involved in ALS
37	45	1	Cluster II	ANKRD1	PPARA activation of gene expression
40	44	5	Cluster II	HDAC9	NOTCH 1 regulation of transcription
48	42	3	Cluster II	ID2	Signaling by Receptor Tyrosine Kinases, NRTKs
49	42	2	Cluster II	ANXA3	Anexin 3 calcium binding phospholipid dependent, ibrain expressed
56	39	4	Cluster II	GATM	Creatine metabolism
73	34	1	Cluster II	FGFR1OP	Mitotic cycle regulation, organelel biogenesis
81	29	0	Cluster II	HID1	vesicle trafficking within the trans-Golgi network, brain development
17	50	2	Cluster III	ONECUT2	Neuronal differentiation TF
18	49	1	Cluster III	SYT4	clathrin-mediated endocytosis
41	47	0	Cluster III	ID1	NGF-/TRK-stimulated transcription
23	47	4	Cluster III	ERBB4	Nuclear signaling by ERBB4, MAPK signaling
51	41	4	Cluster III	SH3GL2	Clathrin-mediated endocytosis, axon guidance
65	37	4	Cluster III	NEUROG2	Neurogenin TF, neuronal differentiation of ectodermal cells .
72	34	1	Cluster III	PCSK2	Insulin processing
78	31	4	Cluster III	MTRNR2L8	Humanin like neuroprotective, antiapoptotic
80	29	2	Cluster III	MTRNR2L4	Humanin Like neuroprotective, antiapoptotic
4	53	1	Cluster IV	MARCKSL1	cell migration, neural tube formation
9	51	1	Cluster IV	FAT4	atypical cadherin 4, neural tube closure
10	51	4	Cluster IV	SORL1	Amyloid fiber formation, metabolism of proteins
9	51	4	Cluster IV	SLC7A11	NFE2L2 regulating anti-oxidant/detoxification enzymes
67	51	3	Cluster IV	CHL1	Axon guidance, regulates chaperone genes
15	50	3	Cluster IV	FOXC1	Neurodevelopmental TF
64	37	1	Cluster IV	SMARCE1	Chromatin modifying enzymes
1	53	2	Cluster V	MYLIP	Downregulation of SMAD2/3:SMAD4 transcriptional activity
3	53	2	Cluster V	PCDH17	Protocadherin, dendritic and spine developmnet
13	50	5	Cluster V	SPP1	Signaling by PDGF, integrins
16	50	5	Cluster V	SKIL	Signaling by TGF-beta Receptor Complex, ECM regulation
24	46	2	Cluster V	SYT10	Transcriptional Regulation by NPAS4
32	46	1	Cluster V	STMN2	RHO GTPase cycle
14	50	0	Cluster VI	BSN	Sensory pathways
21	47	1	Cluster VI	ARC	NGF/NTRKs regulated transcription
82	28	5	Cluster VI	SYT16	synaptotagmin, synapse formation
2	53	6	Cluster VII	DARC	GPCR ligand binding
7	52	2	Cluster VII	LAMA2	ECM proteoglycans
8	52	0	Cluster VII	DKK1	TCF-dependent signaling by WNT lsignaling,
19	49	2	Cluster VII	PCDH19	Protocadherin, dendritic and spine developmnet
25	46	2	Cluster VII	TNC	ECM proteoglycans
27	46	1	Cluster VII	SPARC	ECM proteoglycans
5	52	5	Cluster VIII	TSPAN2	Transpanin, surface glycoprotein, interacts with integrins, regulates cell development
6	52	0	Cluster VIII	SYT11	synaptotagmin, clathrin-mediated endocytosis
26	46	5	Cluster VIII	LRP1	Signaling by WNT
28	46	0	Cluster VIII	PFN1	axon guidance, PCP/CE pathway
31	46	1	Cluster VIII	SPTAN1	Interaction between L1 and Ankyrins, nephrin interactions, apoptosis
34	45	2	Cluster VIII	MACF1	Microtubule-actin cross-linking factor 1
55	40	1	Cluster VIII	SOX8	Neurodevelopmental TF
41	44	1	Cluster IX	NID1	IGF transport anduptake
28	46	2	Cluster IX	FN1	Interleukins and integrins signaling
44	43	1	Cluster IX	NOVA2	Reelin signaling and neuronal migration,
45	43	1	Cluster IX	PAX5	RUNX1 regulates transcription of genes involved in BCR signaling
52	41	2	Cluster IX	PRELID1	Transcriptional Regulation by TP53, apoptosis
75	32	0	Cluster IX	NPAS4	gene expressionregulation by NPAS4, Experience-Induced Target Gene Programs in Neurons
83	28	2	Cluster IX	SLFN13	tRNA, rRNA cleavage, metabolism
39	44	3	Cluster X	ETV5	developmental TF
46	43	2	Cluster X	LRP2	Clathrin mediate endocytosis, metabolsim of steroids
53	41	1	Cluster X	GRID2	NMDA receptor-mediated neuronal transmission
57	39	0	Cluster X	CNTN6	CHL1 cell adhesion interactions
58	39	3	Cluster X	HES1	NOTCH1,2,3,4 signaling
59	39	3	Cluster X	ESYT1	RHO GTPase cycle
61	37	0	Cluster X	SCRIB	RHO GTPase cycle, WNt signaling beta-catenin dependet
62	37	1	Cluster X	NID2	Cell adhesiopn protein, binds collagen
71	34	2	Cluster X	ANKRD13B	development/function of olfactory
74	32	0	Cluster X	MTRNR2L6	humanin like protein, mitochondrial genome, neuroprotective and antiapoptotic factor
43	43	2	Cluster X	IER2	developmental TF
50	41	6	Conectors	WNT7A	WNT biogenessi, trafficking, signaling
30	46	3	Conectors	SYT13	Synaptotagmin, synaps formation
70	34	3	Conectors	SLFN12	Schlafen transmembrain protein - neurodevelopmnet?
38	44	5	Conectors	SYT17	Synaptotagmin - motor neuron protection
67	35	0	Conectors	UCHL1	Deubiquitination

Supplemental Table S3B. NSD genes (131 genes from clustered network in Fig. 5B and circular GAN in Fig. 4B) whose average activity was altered in NCC^{TK}; cluster assignment and number of connections with other genes are listed. The gene functions were assigned based on g:Profiler hsapiens and Reactome.

Gene No network	No Edges NCC	No edges NCCTK	No Edges NPC	NCC cluster	NCC cluster genes	
67	27	3	0	Cluster I	MDGA2	Post-translational modification: synthesis of GPI-anchored proteins
68	21	4	0	Cluster I	NFIB	RNA Polymerase III Transcription Termination
69	20	7	0	Cluster I	COBLL1	Cadherin binding/signaling
70	20	0	28	Cluster I	SLFN13	rRNA, tRNA metabolism
71	20	8	34	Cluster I	FGFR1OP	Cell cycle and mitotic control, centrosme,microtubules
72	20	4	0	Cluster I	ZIC3	Pluripotency TF
73	20	7	0	Cluster I	PTPRD	Receptor-type tyrosine-protein phosphatases
74	20	2	42	Cluster I	ANXA3	GPCR ligand binding
19	38	0	45	Cluster I	ANKRD1	PPARA activates gene expression
77	19	1	0	Cluster I	NRCAM	Regulation of lipid metabolism by PPARalpha
78	19	7	34	Cluster I	SLFN12	SLFN12 pro-apoptotic pathway
79	19	3	0	Cluster I	NMUR2	GPCR ligand binding
80	19	3	0	Cluster I	PPP1R17	Neural progenitor cell-cycle progression
42	34	2	45	Cluster I	ANXA1	Interleukins signaling
83	18	7	0	Cluster I	MDM2	NPAS4 regulates gene expression, TP53 expression, RUN3 mediated transcription
84	18	0	50	Cluster I	FOXC1	Neuropdevelopmental TF
87	17	0	0	Cluster I	AGER	TRAF6 mediated NF-kB activation, LTR cascade, interleukin signaling
96	16	1	0	Cluster I	S100B	signaling by ERBB4, TRAF6 NF-kB activation, interleukin signaling, LT cascade,
105	15	2	0	Cluster I	COL25A1	ECM regulation
112	13	0	0	Cluster I	PPARGC1A	Mitochondria biosynthesis, PPARA activates gene expression, FOXO mediate transcription
122	11	8	39	Cluster I	CNTN6	axon guidance, cell adhesion
85	18	1	0	Cluster II	USH1G	Ankyrin repeat protein, neural, retinal developmnet
91	16	8	45	Cluster II	MACF1	Microtubules crosslinking
92	16	7	0	Cluster II	TENM3	neuronal development, visual system
95	16	7	0	Cluster II	OTX2	Neurodevelopmental TF
98	16	8	0	Cluster II	CHRDL1	Regulation of Insulin-like Growth Factor (IGF) function, BMP signaling
107	14	10	0	Cluster II	DNM3	Axon guidance, L1CAM interactions
110	13	7	46	Cluster II	FN1	Regulation of IGF transport and Interleukins signaling, NRTKs, and MPK signaling
111	13	3	32	Cluster II	NPAS4	NASP4 gene expression and transcriptional function
115	12	9	39	Cluster II	GATM	Metabolism of amino acids and derivatives
116	12	10	0	Cluster II	HCN1	HCN channels
117	12	8	46	Cluster II	SPTAN1	Signaling by Rho GTPases, Miro GTPases and RHOBTB3, NRTKs, and MPK signaling
118	12	7	0	Cluster II	NEUROD4	Neuronal differentiation TF
120	12	1	0	Cluster II	PCDH15	Protocadherin, axon guidance and contacts
124	8	3	0	Cluster II	TFAP2B	TFAP2 (AP-2) family regulates transcription of growth factors and their receptors
127	6	10	0	Cluster II	SLC17A7	associated with the membranes of synaptic vesicles
128	5	2	40	Cluster II	LEF1	WNT signaling of TCF/LEF:CTNNB1, RUNX
129	5	9	0	Cluster II	SPOCK2	ECM protein
76	20	3	51	Cluster III	SLC7A11	NFE2L2 regulating anti-oxidant/detoxification enzymes
88	17	3	0	Cluster III	SCN3B	Axon guidance,
89	16	1	0	Cluster III	ENO3	Gluconeogenesis
90	16	0	35	Cluster III	UCHL1	deubiquiti
93	16	0	0	Cluster III	NRBP2	nuclear receptor binding protein 1, promotes cell death, tumor suppressor
94	16	2	0	Cluster III	ROBO3	Axon guidance, pathfinding ROBO and SLIT
97	16	0	0	Cluster III	DIO3OS	Lnc RNA
90	16	5	51	Cluster III	CHL1	Axon guidance, ubiquitination
100	16	5	46	Cluster III	STMN2	RHO GTPase cycle
101	16	6	0	Cluster III	PCDH9	cell adhesion, protocadherin
102	15	2	0	Cluster III	ARHGAP42	RHO GTPase cycle
103	15	2	47	Cluster III	ERBB4	ERBB2,4 signaling
104	15	7	0	Cluster III	BDNF	Activated NTRK2 signals through RAS
106	15	2	50	Cluster III	SPP1	RUNX3 Regulates Immune Response and Cell Migration
113	13	0	0	Cluster III	NTRK3	BDNF signals through RAS, MAPK
119	12	5	49	Cluster III	SYT4	Clathrin-mediated endocytosis
121	11	5	0	Cluster III	BARHL2	developmental TF
125	7	4	0	Cluster III	NHLH2	developmental TF
126	6	17	0	Cluster III	JAG2	NOTCH1,2,3,4 signaling
69	20	5	0	Cluster III	COBL	Actin binding protein
131	3	0	0	Cluster III	SEMA3D	axon guidance
62	23	0	0	Conectors	DLG2	guanylate kinase, neural developmnet, schizophrenia
66	22	2	43	Conectors	LRP2	WNT signaling
53	27	3	50	Cluster IV	BSN	presynaptic sekelton, synaptogenesis
59	23	6	50	Cluster IV	ONECUT2	developmental TF
61	23	21	53	Cluster IV	PCDH17	WNT signaling
63	22	14	0	Cluster IV	CD97	mitogenic pathway activation,
64	22	7	0	ClusterIV	NEO1	cell surface development controlling protein
34	35	7	0	Cluster V	LHX1	developmental TF
40	34	0	51	Cluster V	FAT4	neuroal tube closure
42	34	20	49	Cluster V	ANXA11	Anexin phospholpiid binding proteins, involved in ALS
44	33	5	46	Cluster V	LRP1	LRP1 and WNT signaling
98	16	9	0	Cluster V	CHRD	BMP, TGF signaling
50	31	15	0	Cluster V	C1QL1	synaptogenesis
1	44	21	0	Cluster VI	ZFP36L1	Ring finger proteins controls cell proliferation and migration
13	40	21	0	Cluster VI	WNT10B	WNT signaling
25	37	22	0	Cluster VI	NDNF	neurotrophic factor
31	35	2	0	Cluster VI	NAV2	neuron navigator
37	35	21	41	Cluster VI	WNT7A	WNT signaling
33	35	6	0	Cluster VI	NKD1	WNT signaling
35	35	6	0	Cluster VI	TRIM71	E3 ubiquitin-protein ligase, ESC
46	32	16	0	Cluster VI	ZIC1	neurodevelopmental TF
54	27	1	0	Cluster VI	PRKCH	PKC signaling
57	25	15	0	Cluster VI	RARG	Retinoic Acid signaling
60	23	4	51	Cluster VI	SORL1	processing of the amyloid-beta (Abeta) precursor protein (APP)
65	22	6	0	Cluster VI	SULF2	HSPG regulator

Gene No network	No Edges NCC	No edges NCCTK	No Edges NPC	NCC cluster	NCC cluster	Functions in corresponding order
29	35	6	0	Cluster VII	CBLN1	Syanptic organizer protein
39	35	24	0	Cluster VII	GSN	Apoptosis, amyloid fiber formation
36	34	21	0	Cluster VII	CSPG5	Glycosaminoglycan metabolism
4	42	0	0	Cluster VIII	KLHL17	regulation of actin-based neuronal function
5	41	4	0	Cluster VIII	MYT1L	Neurodevelopmentl TF
6	41	22	0	Cluster VIII	PRKCG	PKC signaling, neural developmnet
7	41	23	0	Cluster VIII	SALL1	neurogenesis, brain cortex
17	39	2	0	Cluster VIII	FZD10	WNT sigaling
26	37	5	49	Cluster VIII	PCDH19	Cell communication, adhesion in the nervous system
7	43	23	0	Cluster IX	ATP1A3	Ion transport by P-type ATPases
3	43	7	34	Cluster IX	EGR2	EGR2 and SOX10 in Schwann cell myelination, NRTK signaling, activation of HOX genes
19	38	1	34	Cluster IX	ANKRD13B	Ankyrin repeat domain protein/receptor internalization
47	31	4	37	Cluster IX	ANKRD18DP	Nuclear protein in TNF signaling
9	41	1	46	Cluster X	PFN1	actin binding, cytoskeleton control
10	41	9	0	Cluster X	SEMA6B	Semaphorin axon guidance
11	41	1	0	Cluster X	SOX3	developmental TF
12	41	22	0	Cluster X	MYCL1	developmental TF
16	40	13	0	Cluster X	PRDM12	developmental TF
18	39	21	0	Cluster X	EFNB3	ephrin axon guidance
14	40	1	0	Cluster XI	APOE	Nuclear signaling by ERBB4
15	40	6	0	Cluster XI	KIF17	RhoA-GTPase signaling to influence junctional actin, NMDA receptors assembly
21	38	22	0	Cluster XI	BAI2	cell adhesion
23	38	4	43	Cluster XI	IER2	Immediate Early Response TF IER2
27	37	1	0	Cluster XI	CNTN2	axonal guidance - NrCAM interactions
28	36	18	0	Cluster XI	DIO3	Regulation of thyroid hormone activity
8	41	2	0	Cluster XII	PAX7	**Neurodevelopmental TF**
20	38	10	0	Cluster XII	MDGA1	neuronal migration is involved in SZ pathology
22	38	24	0	Cluster XII	NHLH1	Developmental TF
24	38	4	0	Cluster XII	MYCN	TF, Signaling by Receptor Tyrosine Kinases
38	34	18	0	Cluster XII	PTCH1	Hedgehog signaling
39	34	24	40	Cluster XII	SOX8	developmental TF
41	34	23	0	Cluster XII	WNT7B	Signaling by WNT
45	33	1	0	Cluster XII	WNT9B	Signaling by WNT
55	27	26	0	Cluster XII	WNT1	Signaling by WNT
58	25	23	0	Cluster XII	S1PR5	GPCR downstream signalling
82	18	4	0	Cluster XII	SLC45A3	transmembrane transport
109	13	4	0	Cluster XII	SSPO	protein metabolism
12	41	2	0	Cluster XII	MYC	Signaling by WNT, Binding of TCF/LEF:CTNNB1 to target gene promoters, NOTCH, SMAD2/SMAD3:SMAD4 signaling
123	10	2	0	Cluster XII	RTN1	reticulon - ER trafficking
						**CONNECTORS**
62	23	0	0	Conectors	DLG2	POSTSYNAPTIC Density Protein, associated with SZ
66	22	2	43	Conectors	LRP2	WNT signaling
37	34	3	0	Conectors	CHAC1	Glutathione synthesis and recycling
43	34	4	35	Conectors	OTX1	Neurodevelopmental TF
48	31	1	0	Conectors	MSX1	Neurodevelopmental TF
51	30	20	0	Conectors	CRABP2	Signaling by Retinoic Acid
52	28	14	0	Conectors	EFNA3	ephrin axon guidqance
56	27	15	0	Conectors	CD9	Response to cytosolic Ca2+
86	17	5	46	Conectors	LRFN1	synaptoc adhesion
108	14	23	0	Conectors	SOX9	Neurodevelopmental TF
62	23	0	0	Conectors	DLG2	POSTSYNAPTIC Density Protein, associated with SZ
66	22	2	43	Conectors	LRP2	WNT signaling

Supplemental Table S3C. NSD genes (90 genes (from clustered network in Fig. 5C and circular GAN in Fig. 4C) whose average activity was altered in NCC^{NLS}; cluster assignment and number of connections with other genes are listed. The gene functions were assigned based on g:Profiler hsapiens and Reactome.

Gene No	Edge count		Gene name	CLUSTER	Pathway/function
68	8		NID1	I	Extracellular matrix organization
69	8		HCN1	I	HCN channels
72	8		SYT13	I	synaptotagmin, synapse formation/function
73	8		ID2	I	NGF-stimulated transcription, signaling by NTRK
74	8		NR4A2	I	Transcription regulation by RUNX1,
75	8		INA	I	internexin, morphogenesis of neurons
85	6		S100B	I	Nuclear signaling by ERBB4
41	19		PRELID2	II	PRELI domain containing 2 protein nvolved in embryonic development., phospholipid transfer
48	13		MYC	II	MAPK6/MAPK4 signaling, TGF Beta, Wnt signaling
49	13		SEZ6L2	II	promotes caspase-dependet aopotosis
50	12		CDK5R2	II	NGF regulated transcitpton, Signaling by NTRKs
51	12		RTN1	II	Reticulon, endoplasmic neuroendocrine secretion
52	12		MAPT	II	Apoptotic cleavage of cellular proteins
54	12		PRDM8	II	Histone methyltransferase-neuronal cicuit assembley
55	12		NID2	II	axon guidance, Laminin interactions, ECM processing
56	10		PCSK2	II	insulin processing
57	10		OTX2	II	brain,neurodevelopmental TF
59	9		PRKCH	II	GPCR downstream signalling
60	9		PCDH15	II	Protocadherin, axon guidance, cell adhesion
86	5		ID4	II	NGF regulated transcitpton, Signaling by NTRKs
87	5		ISLR2	II	axon guidance extension
89	4		GRID2	II	glutamate synaptic transmission
80	7		DLG2	connector	POSTSYNAPTIC Density Protein, associated with SZ
90	3		SYT16	II	synaptotagmin, synapse formation/function
44	15		SOX9	III	Neurodevelopmental TF
46	13		TFAP2B	III	TFAP2 (AP-2) family regulates transcription of growth factors and their receptors
47	13		SNPH	III	Mitochondrial function regulation
53	12		TNFRSF21	III	TNF receptor, apoptosis
61	9		KLHL15	III	adaptors for ubiquitin ligases, involved in intelectual disability
62	9		UCHL1	III	deubiquitination, proteases
63	9		CNTN1	III	formation of axon connections in the developing nervous system
64	8		THRB	III	thyroid hormone receptor, developmnet of the nervous system
65	8		SYT10	III	Transcriptional Regulation by NPAS4
66	8		SCN2A	III	voltage-gated sodium channel, propagation ofaction potential
67	8		ANKRD1	III	PPARA activates gene expression
70	8		PRELID1	III	Transcriptional Regulation by TP53, apoptosis
71	8		NMUR2	III	Neuromedin receptor, synaptic transmission
76	7		FGFR1OP	III	Cell cycle and mitotic control, centrosme,microtubules
77	7		SYT4	III	Clathrin-mediated endocytosis
78	7		BARHL2	III	developmental TF
81	7		STMN2	III	RHO GTPase cycle
82	7		CHL1	III	deubiquitination, proteases
83	7		COL25A1	III	ECM regulation
84	6		EID1	III	P300 binding differentiation inhibitor
2	38		MYCL1	IV	developmental TF
3	37		HES1	IV	NOTCH1,2,3,4 signaling
5	37		FOXB1	IV	neurodevelopmental TF
	37		INSM1	IV	neurodevelopmental TF
8	36		SEMA3F	IV	semaphorin, axon guidance
9	36		CEBPB	IV	neurodevelopmental TF
10	36		FOS	IV	neurodevelopmental TF
11	36		PAX7	IV	neurodevelopmental TF
12	36		BAI1	IV	postsynaptic adhesion-G protein-coupled receptor, synaptogenesis
13	13		FOSCL2	IV	neurodevelopmental TF
14	35		DLL3	IV	NOTCH ligand
15	35		NR4A3	IV	neurodevelopmental TF, cell migration
17	35		SOX3	IV	developmental TF
18	34		ESYT1	IV	extended synaptotagmin
19	34		MYCN	IV	TF, Signaling by Receptor Tyrosine Kinases
21	33		MYT1L	IV	Neurodevelopmentl TF
22	33		KLHL17	IV	regulation of actin-based neuronal function
24	32		CNTN2	IV	axonal guidance - NrCAM interactions
25	31		GSX1	IV	Neurodevelopmentl TF
26	31		OTX1	IV	Neurodevelopmental TF
28	31		LHX5	IV	control dendritogenesis and spine morphogenesis, Cajal-Retzius cell developmnet
29	31		CPLX2	IV	complexin, synaptic vesicle exocytosis
30	30		ID1	IV	DNA binding inhibitor
32	30		FOSL1	IV	Neurodevelopmentl TF
33	30		CHAC1	IV	Glutathione synthesis and recycling
35	29		LY6H	IV	regulates acetylcholine Nicotinic receptor transmission
37	24		NKD1	IV	WNT signaling
38	21		IRX5	IV	Neurodevelopmentl TF
39	20		OTP	IV	Neurodevelopmentl TF
42	18		LHX1	IV	developmental TF
43	15		ID3	IV	controls of DNA binding of TF
45	14		HID1	IV	vesicle trafficking within the trans-Golgi network, brain development
1	38		SYT17	connector	Synaptotagmin - motor neuron protection
4	37		PRDM12	connector	developmental TF
7	37		TIMP2	connector	inhibitor of metalloproteinase, promotes neuronal differentiation
16	35		UNCX	connector	Neurodevelopmentl TF, developmental TF
20	34		ANKRD13B	connector	Ankyrin repeat domain protein/receptor internalization
23	32		SRF	connector	Neurodevelopmentl TF, stimulates both proliferation and differentiation
27	31		SYT11	connector	synaptotagming, inhibits clathrin-mediated and bulk endocytosis
31	30		APC2	connector	stabilizes microtubules, regulates neuronal migration, axon guidance
34	30		SCRT1	connector	developemntal TF, regulates cell proliferation
36	26		ANKRD18DP	connector	nuclear protein in TNF signaling
40	19		DTX1	connector	deltex, regulates NOTCH pathway
58	10		DNM3	connector	Axon guidance, L1CAM interactions
79	7		KLHL1	connector	regulation of actin-based neuronal function
88	4		SDC4	connector	syndecan, receptor in itracellular signaling, exosome biogenesis

Supplemental Table S4. Changes in positive coordination of functionally related genes during NPC→NCC transition and by inhibition or overexpression of active nFGFR1; 0 indicates absence of positive correlation. Two left columns list pairs of correlated genes

Table S4A. Coordinated NPC genes affected by reduced nFGFR1 function or differentiation to NCC.

		NCC	NPCTK-	NCC
FGF19	ANKRD1	0.995292	0	0
FGF19	DDIT4	0.993844	0	0
FGF19	EGR2	0.240346	0.994076	0
HES1	CNTN6	0.999919	0	0
HES1	ESYT1	0.999996	0	0
HES1	ETV5	0.897844	0.995465	0

Commentary: Table shows examples of NPC interactions affected by differentiation to NCC and by dominant negative nuclear FGFR1(SP-/NLS)(TK-). The significant correlations of HSE1 with ESYT1 (extended synaptotagmin) and CNTN6 (contactin 6), found in NPCs, were absent in NCCs and in NPCs with reduced nFGFR1 function. The significant correlations of mitogenic FGF19 and developmental genes ANKRD1, DDTIT4, and FAT4 were similarly absent in NCCs and NPCs with reduced nFGFR1, indicating that these pairs of genes were synchronized in NPCs by nFGFR1. By contrast, HES1 was significantly correlated with ETV5 only in NPCs with reduced nFGFR1 function, indicating that the two genes are "desynchronized" by endogenous nFGFR1 in NPCs.

Table S4B. Coordinated NPC genes affected by reduced nFGFR1 function.

		NCC	NCCTK-	NPC
FZD10	ATP1A3	0.991213	0	0
FZD10	CBLN1	0.998166	0	0
FZD10	CSPG5	0.996417	0	0
FZD10	EGR2	0.988015	0.348458	0
FZD10	WNT10B	0.99998	0	0
FZD10	WNT7A	0.997362	0	0
ROBO3	NRBP2	0.99996	0	0
ROBO3	PCDH9	0.973449	0.633073	0
PAX7	ANKRD13B	0.986944	0	0
PAX7	APOE	0.995916	0	0
PAX7	ATP1A3	0.989484	0	0
PAX7	BAI2	0.990258	0	0
PAX7	KIF17	0.997853	0	0
PAX7	MYCL1	0.997027	0	0
PAX7	MYCN	0.992485	0	0
PAX7	NHLH1	0.989979	0	0
PAX7	OTX1	0.974116	0	0
MYCL1	APOE	0.985997	0	0
MYCL1	ATP1A3	0.997688	0.991143	0
MYCL1	BAI2	0.976584	0.989664	0
MYCL1	EFNB3	0.982794	0.986289	0
MYCL1	EGR2	0.998956	0	0
MYCL1	IER2	0.977673	0	0
MYCL1	KIF17	0.98984	0.911688	0
MYCL1	KLHL17	0.984546	0	0
TRIM71	ANXA11	0.997906	0	0
TRIM71	C1QL1	0.990077	0	0
TRIM71	CBLN1	0.989532	0	0
TRIM71	CHRD	0.991742	0	0
TRIM71	CSPG5	0.992722	0	0
TRIM71	FAT4	0.995939	0.73201	0
TRIM71	LHX1	0.999064	0.991063	0
TRIM71	LRP1	0.995385	0.997175	0
TRIM71	NDNF	0.996439	0	0
TRIM71	NKD1	0.997749	0.999994	0
TRIM71	PCDH19	0.996803	0.999807	0

Commentary: Table lists exemplary interactions between FZD10 (Frizzled receptor for WNT growth factors) and genes for its ligands (WNT7a and WNT10b). In NCCs, FZD10 expression was highly coordinated with that of several neurodevelopmental genes, such as EGR2 (a transcription factor involved in hindbrain development), CBLN1 (involved in cerebellum development), CSPG5 (proteoglycan that functions as a neural growth and differentiation factor), and ATP1A3 (encoding the Na^+/K^+ ATPase sodium pump involved in maintaining the resting potential of the neuronal cell membrane). By contrast, the coordination was absent in NPCs and in NCCs differentiated in the presence of reduced nFGFR1 function.

The significant coordination of ROBO3 (Roundabout Guidance Receptor 3, which controls axon outgrowth fasciculations) and NRBP2 (Nuclear Receptor Binding Protein 2, involved in neural stem/progenitor cell differentiation) as well as PCDH9 (protocadherin, the mutation of which is associated with autism) was present in NCCs but abolished by reduced nFGFR1 function.

In NCCs, expression of PAX7 (transcription factor, broadly involved in neural development) was significantly coordinated with that for a number of other neurodevelopmental genes, including EGR2, KLF174, MYCN, MYTL1, NHLH1, and OTX1 (homeobox gene involved in development of dopamine and serotonin neurons). This coordination was not observed in NPCs or in NCCs that differentiated in the presence of reduced nFGFR1 function.

The significant coordinated activities of MYCL1 (major development-controlling transcription factor) in NCCs were absent in NPCs and some interactions were absent when nFGFR1 function was reduced.

Given the established link between mitochondrial dynamics and neuronal development, it is notable that the expression of TRIM71 (regulator of mitochondrial miRNA and biogenesis) was highly coordinated with that of several neurodevelopmental genes. Many of these correlations were absent in NPCs and thus specific to the neuronal state. Coordination of TRIM71 with ANAXA11, C1QL1, CBLN1, CHRD, CSPG5, and

NDNF (a secreted neurotrophic factor that can promote neuron migration, growth, and survival as well as neurite outgrowth) was not detected in NCCs that differentiated in the presence of reduced nFGFR1 function.

Table S4C. Coordinated NCC genes affected by overexpression of constitutively active nFGFR1.

		NCC	NCCNLS				NCC	NCCNLS
SEMA3F	APC2	0.9872	0.97744		MYCL1	APC2	0.976409	0
SEMA3F	BAI1	0.990442	0		MYCL1	BAI1	0.996729	0
SEMA3F	CEBPB	0.977969	0		MYCL1	CEBPB	0.988345	0
SEMA3F	CHAC1	0.923065	0		MYCL1	CHAC1	0.943635	0
SEMA3F	CNTN2	0.952447	0		MYCL1	CNTN2	0.968376	0
SEMA3F	CPLX2	0.929456	0		MYCL1	DLL3	0.99092	0
SEMA3F	DLL3	0.981561	0		MYCL1	ESYT1	0.992323	0.981012
SEMA3F	ESYT1	0.997788	0		MYCL1	FOS	0.993881	0
SEMA3F	FOS	0.985895	0		MYCL1	FOSL1	0.921986	0
SEMA3F	FOSL2	0.980966	0		MYCL1	FOSL2	0.9905	0
SEMA3F	FOXB1	0.996331	0		MYCL1	FOXB1	0.999602	0
SEMA3F	HES1	0.993574	0.926788		MYCL1	GSX1	0.915586	0.971115
SEMA3F	ID1	0.971737	0		MYCL1	HES1	0.998435	0
SEMA3F	INSM1	0.99801	0.995259		MYCL1	INSM1	0.999984	0
SEMA3F	IRX5	0.948407	0					
SEMA3F	KLHL17	0.992979	0		SOX3	ANKRD13B	0.992494	0
SEMA3F	LHX1	0.924197	0		SOX3	BAI1	0.999374	0
SEMA3F	LHX5	0.903294	0		SOX3	CEBPB	0.999333	0
SEMA3F	LY6H	0.981159	0.999177		SOX3	CNTN2	0.990785	0.909917
SEMA3F	MYCL1	0.998349	0.909952		SOX3	DLL3	0.999828	0
SEMA3F	MYCN	0.967086	0.967196		SOX3	FOS	0.999984	0
SEMA3F	MYT1L	0.992096	0.965384		SOX3	FOSL2	0.999766	0
SEMA3F	NKD1	0.960641	0.938104		SOX3	FOXB1	0.996123	0
SEMA3F	NR4A3	0.984528	0		SOX3	HES1	0.99818	0.93652
SEMA3F	OTP	0.937733	0		SOX3	INSM1	0.993882	0.997508
SEMA3F	OTX1	0.934971	0		SOX3	MYCL1	0.993245	0
SEMA3F	PAX7	0.990955	0		SOX3	MYCN	0.996515	0.960041
SEMA3F	PRDM12	0.995545	0		SOX3	NR4A3	0.999997	0
					SOX3	OTX1	0.982221	0
RTN1	CDK5R2	0.967765	0		SOX3	PAX7	0.999233	0
RTN1	MAPT	0.999628	0		SOX3	PRDM12	0.996854	0
RTN1	MYC	0.989029	0		SOX3	SEMA3F	0.98494	0.999641
RTN1	NID2	0.986684	0		SOX9	MYC	0.991929	0
RTN1	PCSK2	0.957925	0		SOX9	NID2	0.993732	0
RTN1	PRDM8	0.999364	0		SOX9	PRELID2	0.995447	0
RTN1	PRELID2	0.932017	0		SOX9	SEZ6L2	0.994291	0
					SOX9	SNPH	0.999804	0

Commentary: The coordination of SEMA3F (Semaphorin 3F) with RTN1 (membrane trafficking in neuroendocrine cells) and transcription factors MYCL1, SOX3 and SOX9 was not observed with overexpression of nFGFR1. Given that both decreased and increased nFGFR1 signaling disrupts the formation of highly coordinated gene networks, we conclude that the endogenous nFGFR1 levels dictate the formation of the coordinate gene networks in NCCs.

Results of FANMOD*(44)* analysis: significantly overrepresented RCM modules – the module number is followed by its entropy.

<u>Node 3</u>

238 – 0.9183

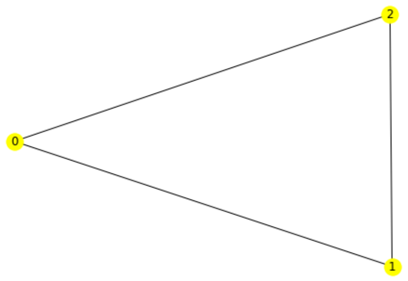

<u>Node 4</u>

31710 – 0.8113

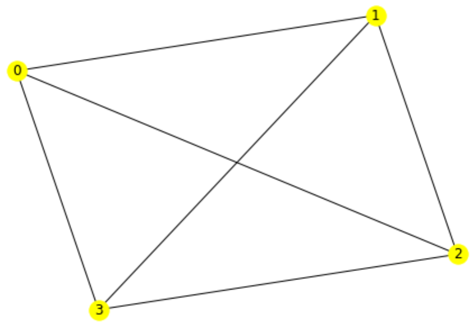

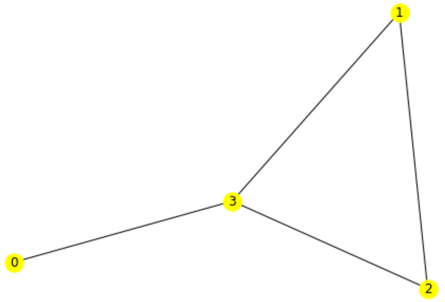

13278 – 0.9544

4958 - 1

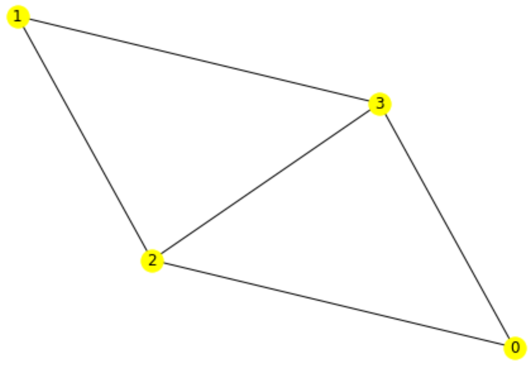

8598 – C – Node 4 – 0.9544

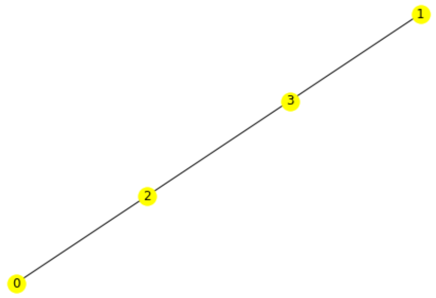

Node 5

8948910 - 0.9710

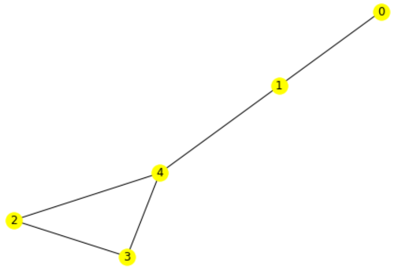

16510910 – 0.7219

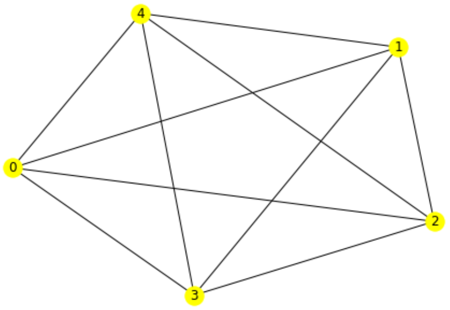

1256886 – 0.9988

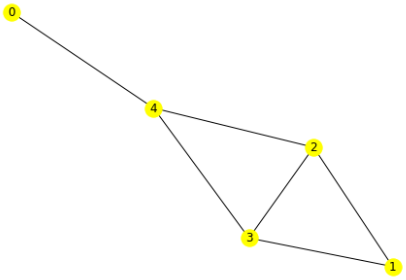

3387326 – 0.7219

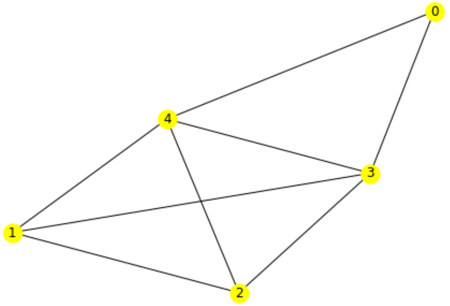

1289662 – 0.9896

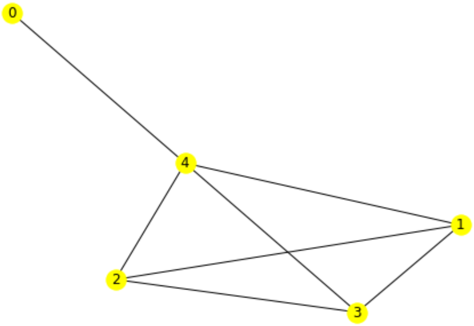

5361086 – 0.9896

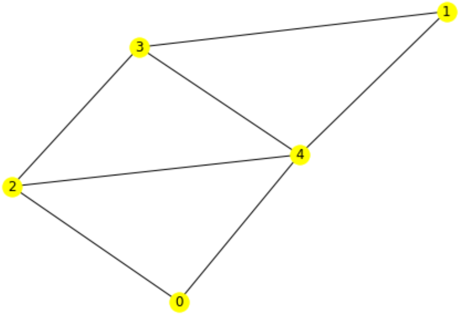

7598014 - 0.8555

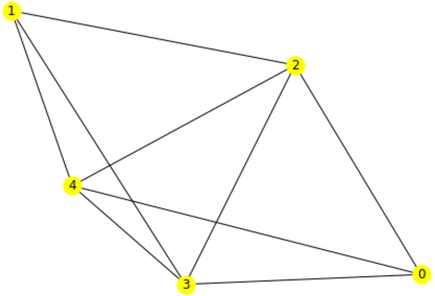

9997502 – 0.9988

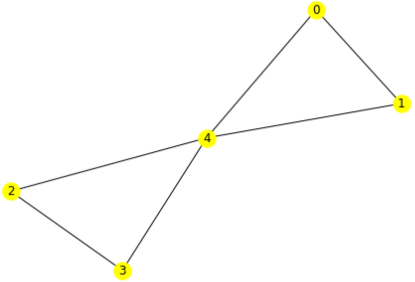

2133644 – 0.9044

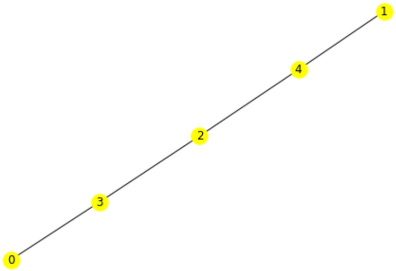

Node 5 – C- (Slide 5)

1084606 – 0.9710

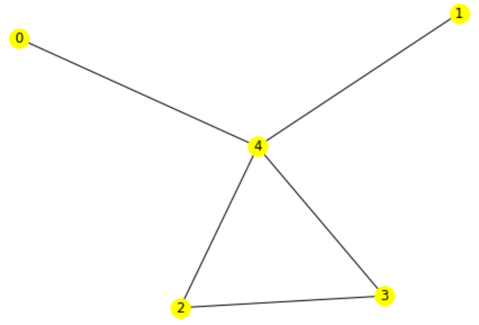

1150398 – 0.9988

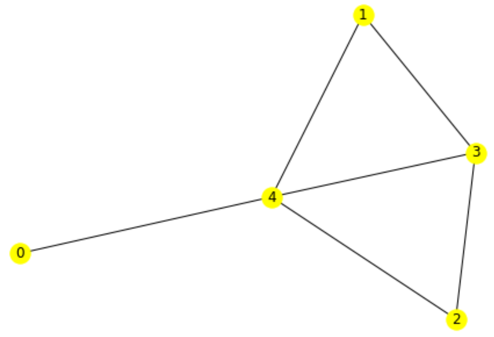

2133678 – 0.9710

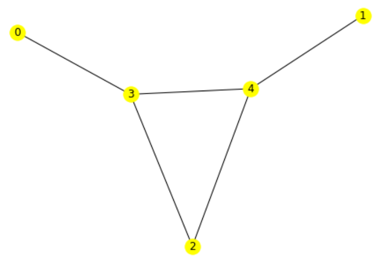

3248062 – 0.9896

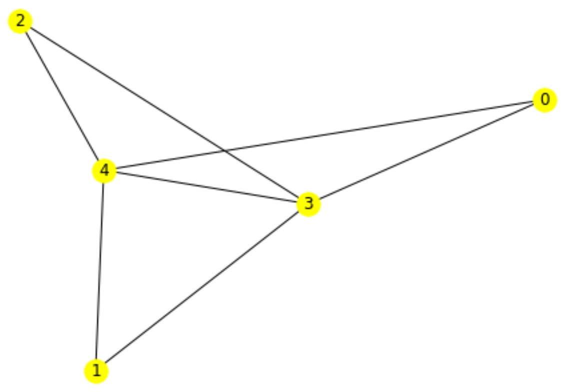

13190438 – 0.9710

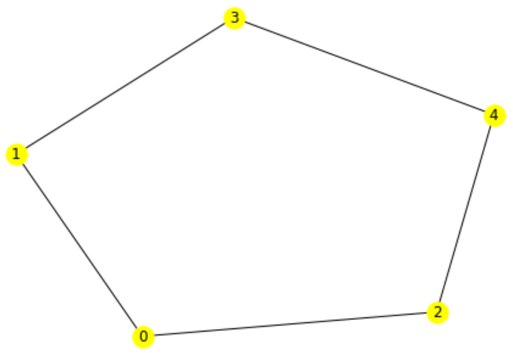

14273982 – 0.9427

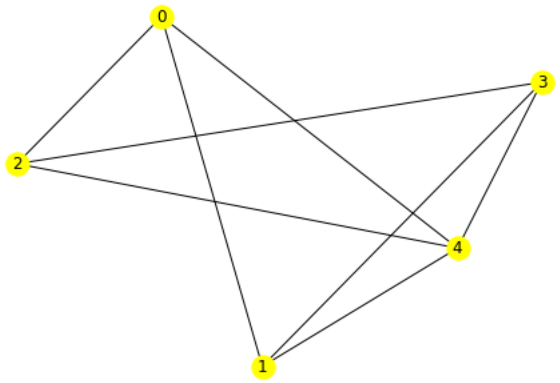

1150398 – 0.9988

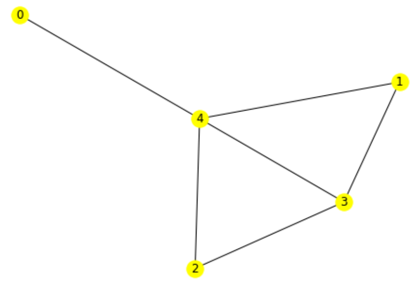

2133678 – 0.9710

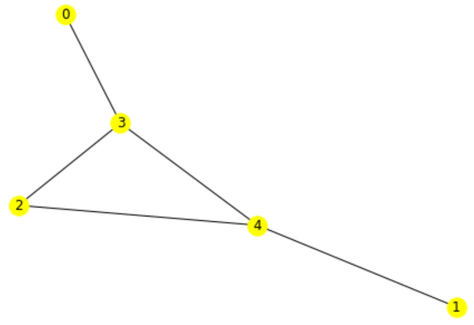

3248062 – 0.9896

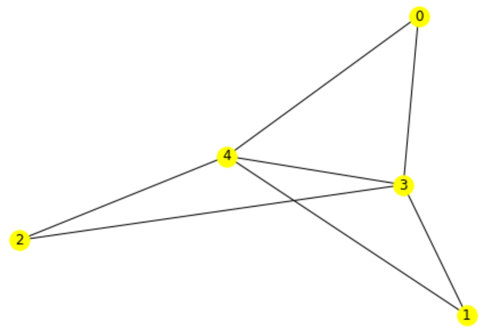

5361086 – 0.9896

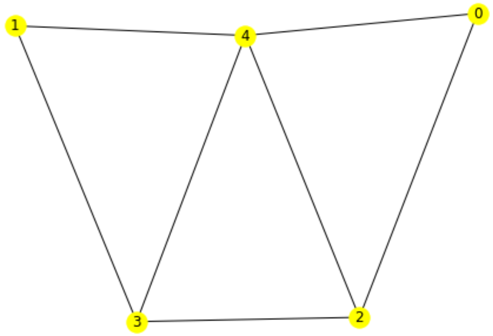

14273982 – 0.9427

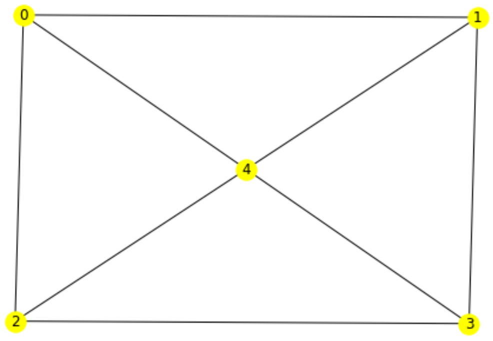

Node 6

17735398238 – 0.9911

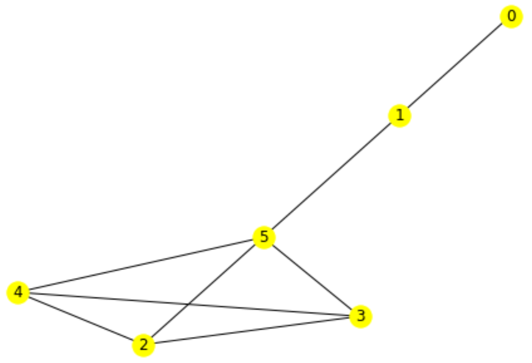

17735136086 – 0.9641

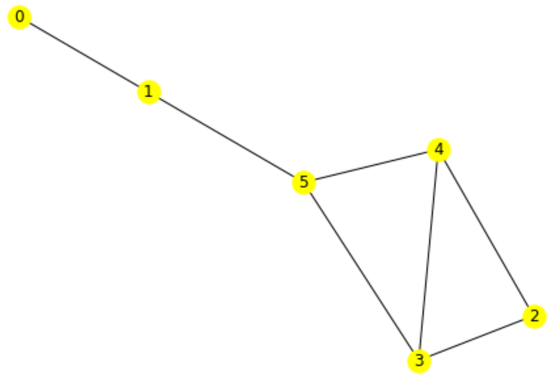

7774125950 – 0.8524

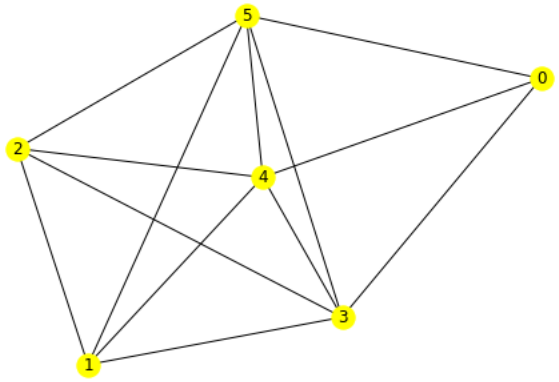

34089189246 – 0.65

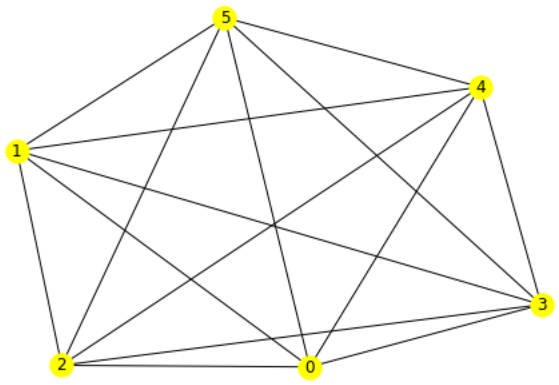

2199165980 – 0.9183

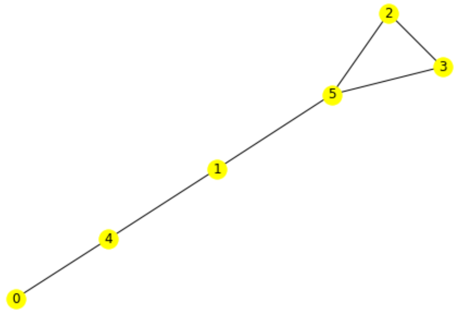

17768953694 – 1.00

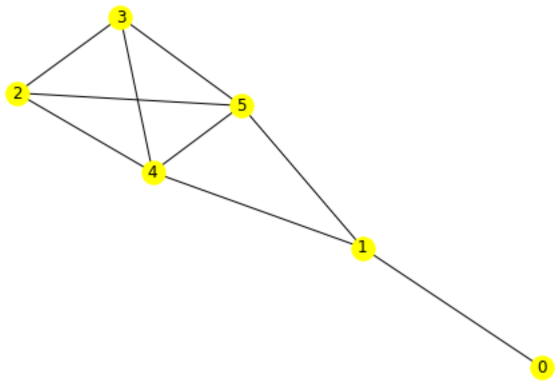

1314764654 – 0.9911

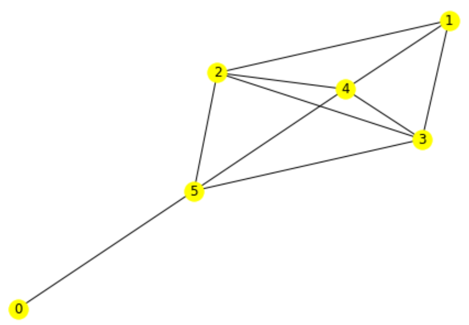

3479027582 – 0.9183

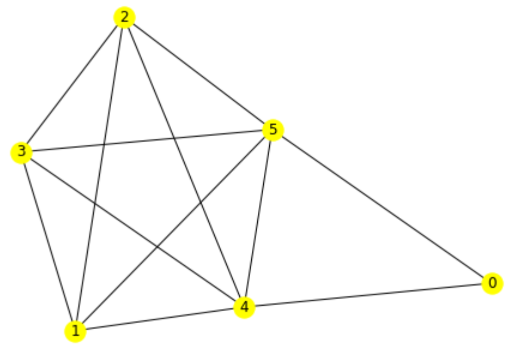

1331541886 – 0.9641

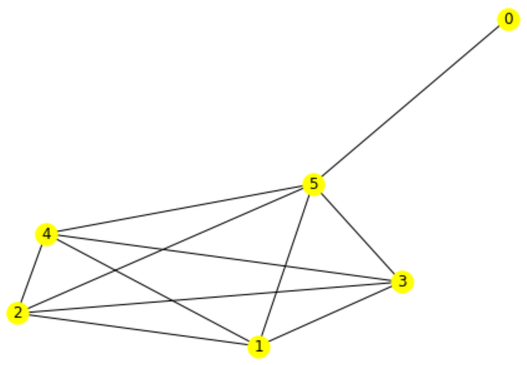

16372449150 – 0.7642

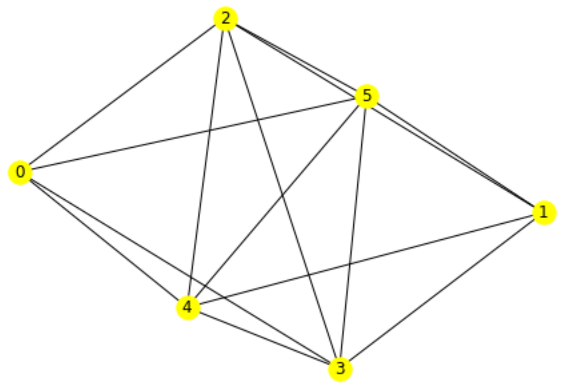

9791453054 – 0.9641

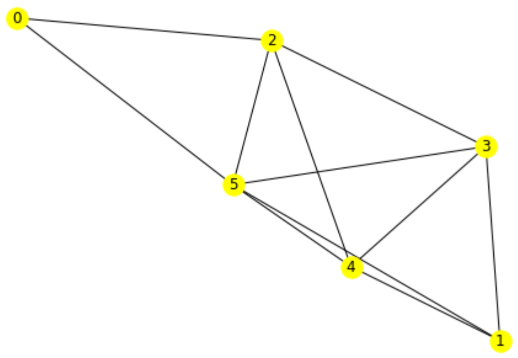

2165873172 – 0.8524

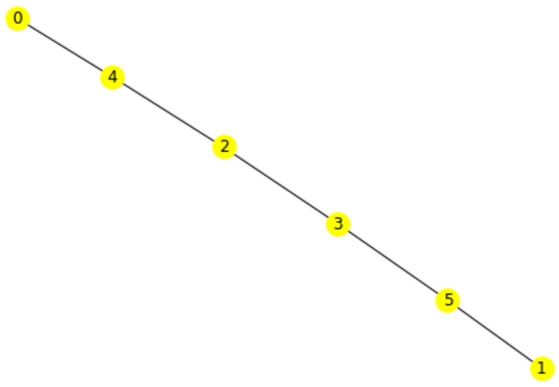

18808877942 – 0.9911

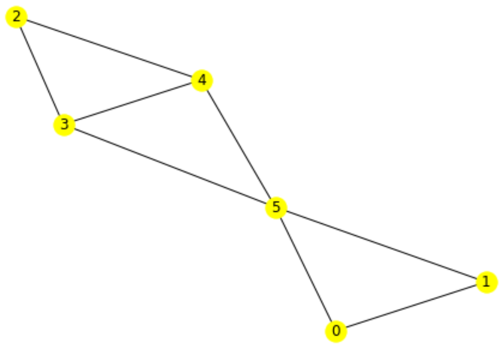

2286172494 – 0.9183

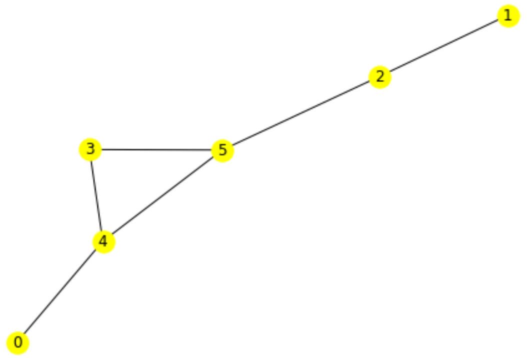

19899128910 – 0.9641

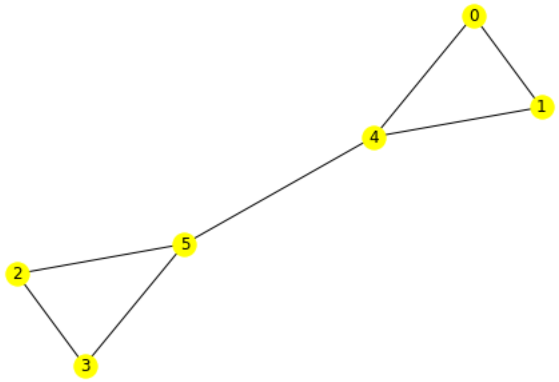

18842695550 – 0.9911

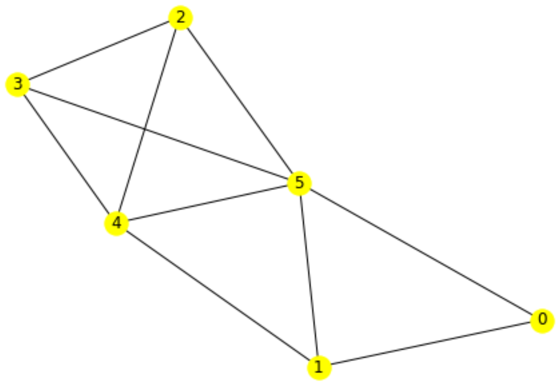

8650011998 – 0.9911

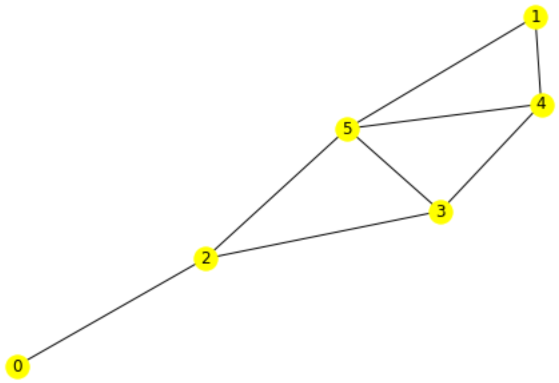

2166143772 – 0.9641

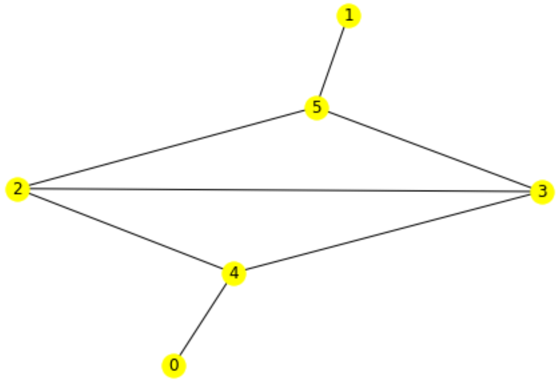

11938938750 – 0.9183

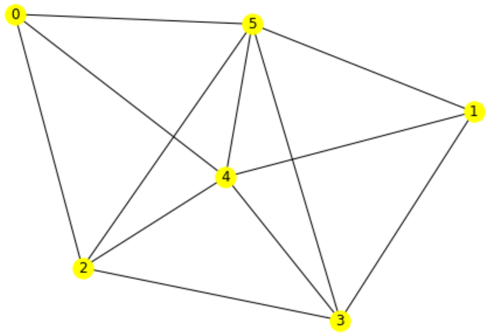

12945635166 – 0.9911

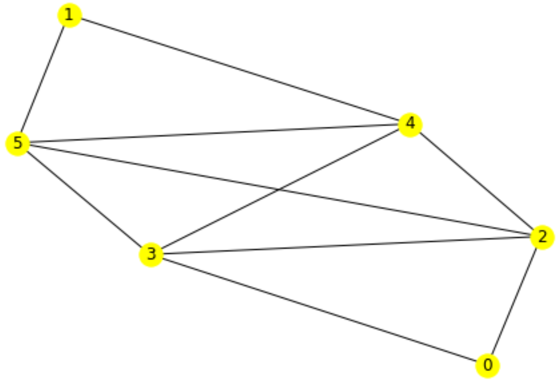

4347311966 – 1

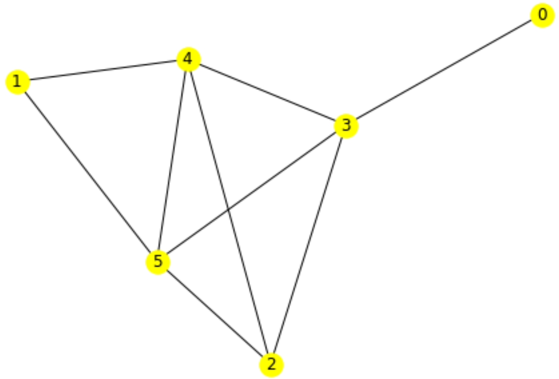

10830560094 – 1

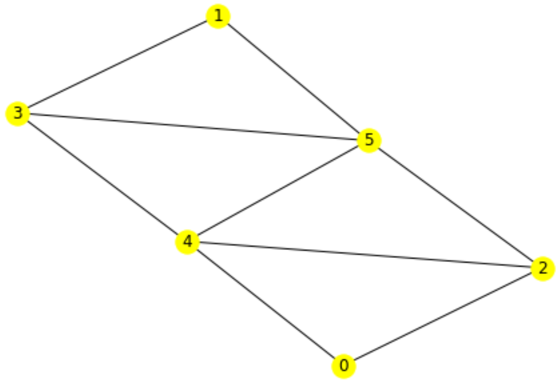

18809140094 – 1

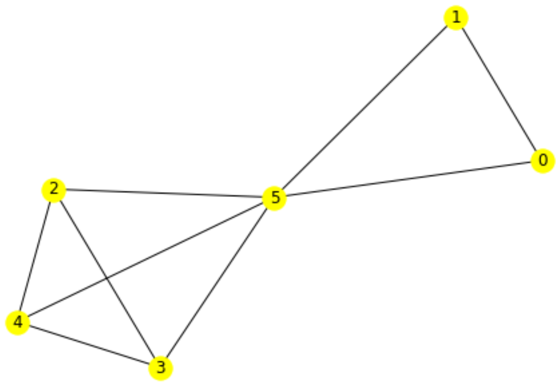

20990181246 – 0.9641

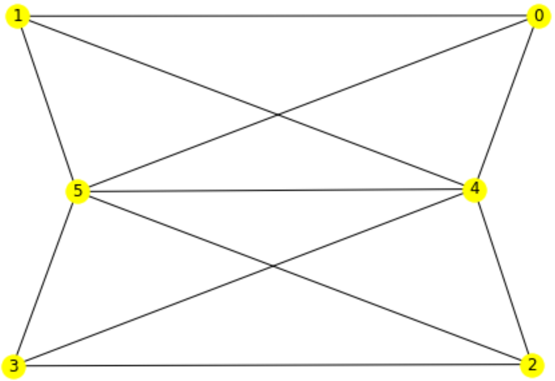

2166143838 – 0.9911

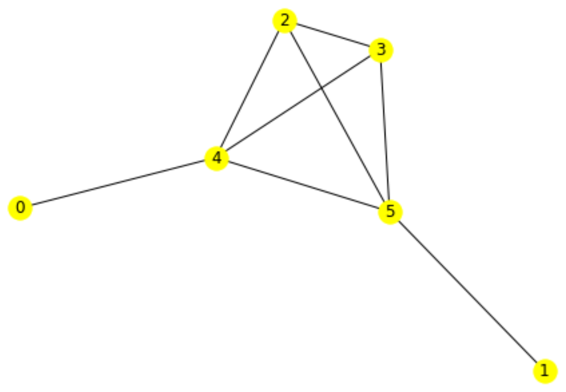

2199166046 – 0.9641

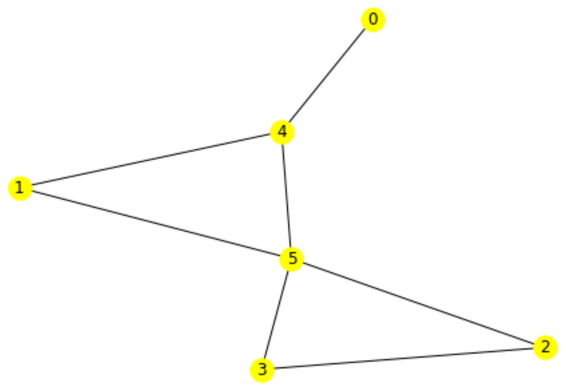

1212428654 - 0.9183

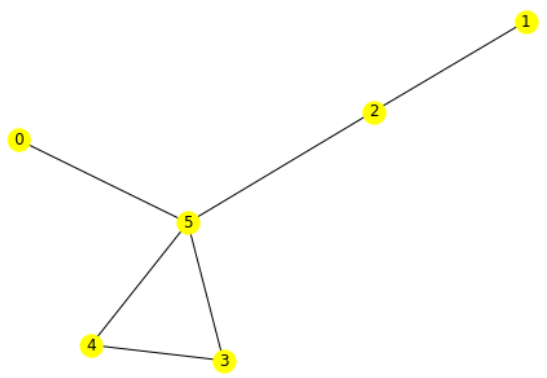

2182388814 - 0.9183

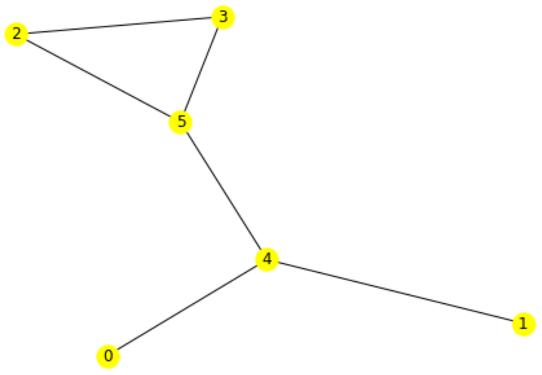

1141715822 - 0.9641

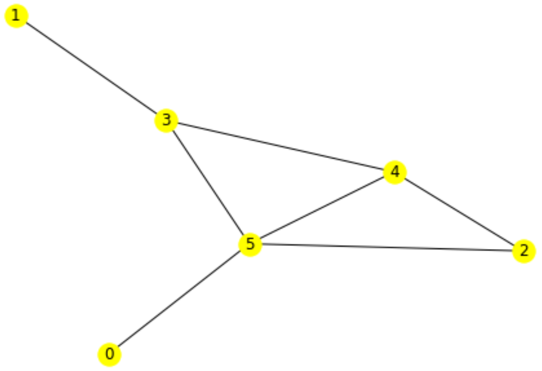

17734316894 - 0.9641

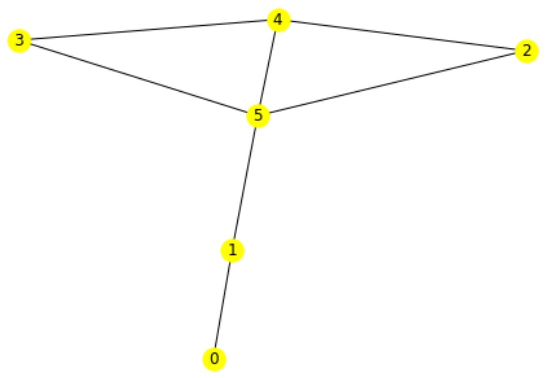

1091056502- 0.9183

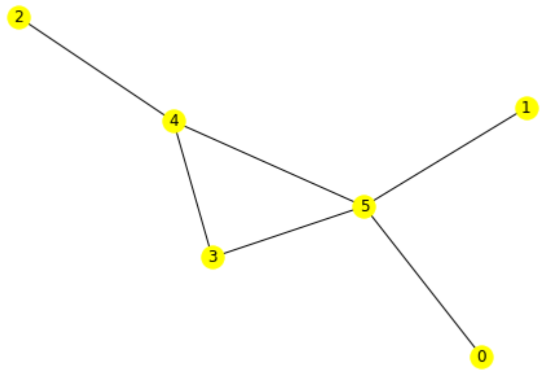

2232236894 - 0.9911

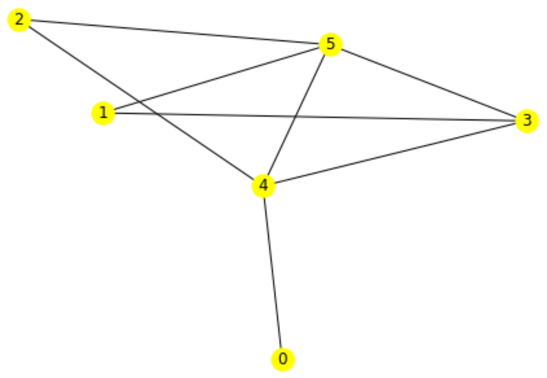

1092137846 - 0.9641

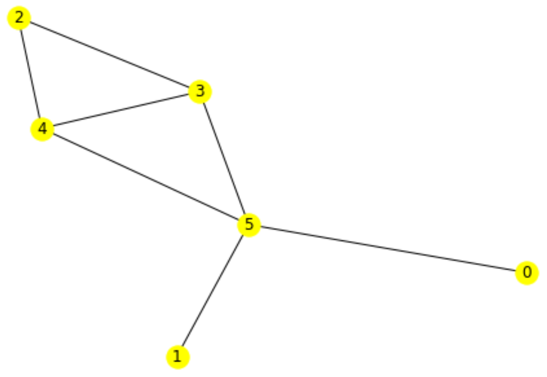

1125955454 - 1

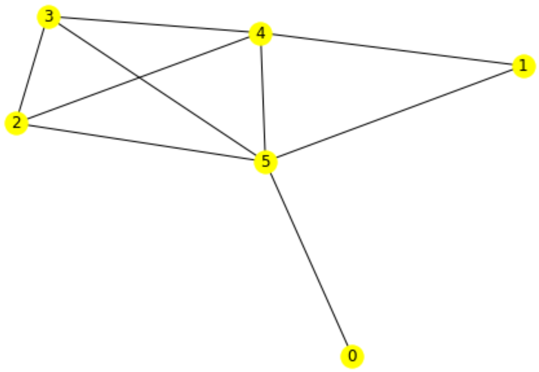

4379845466 - 0.9641

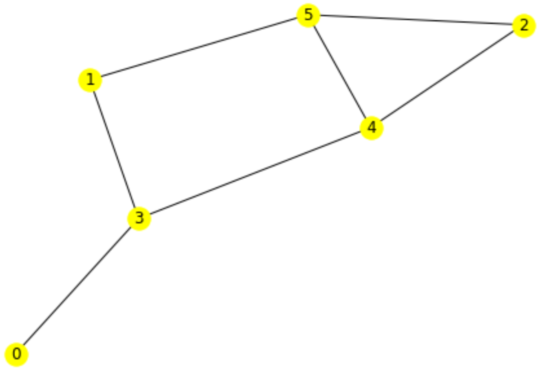

4346230622- 0.9911

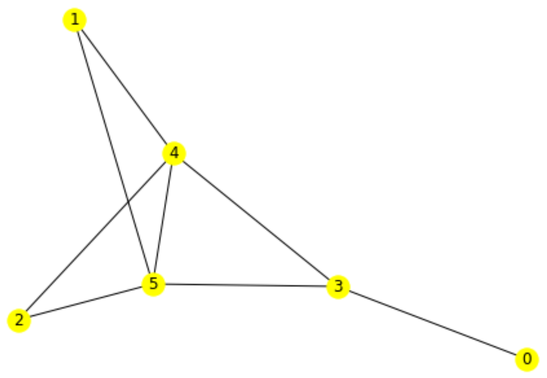

1229205886 - 0.9641

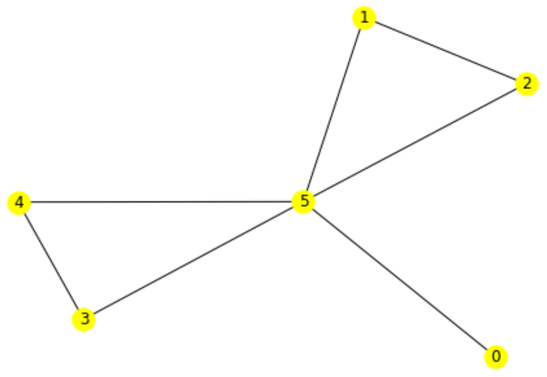

1193129854- 0.9911

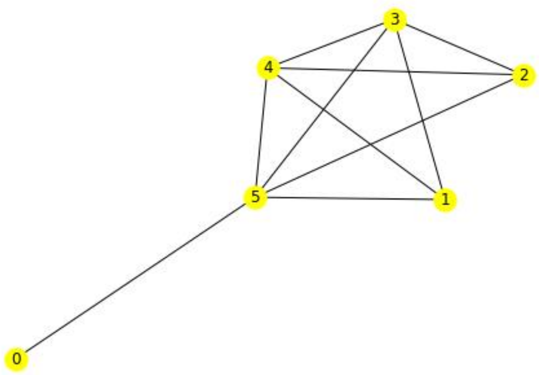

4328928590 - 0.9183

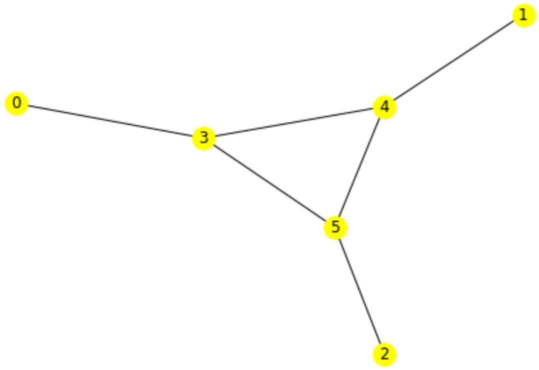

3272907902 - 0.9911

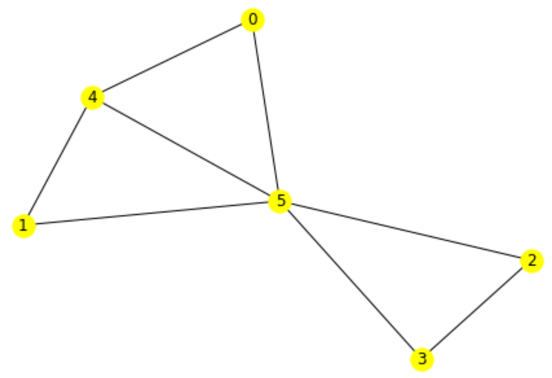

1158488954 - 0.9641

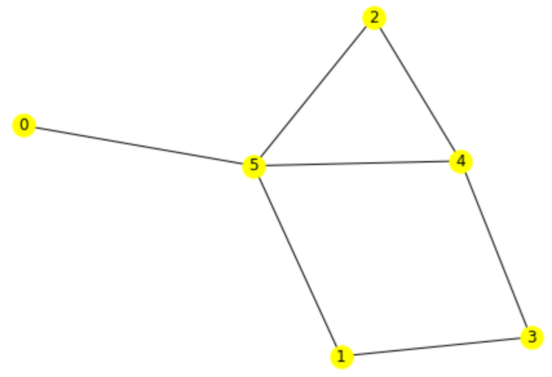

1158493054 - 0.9911

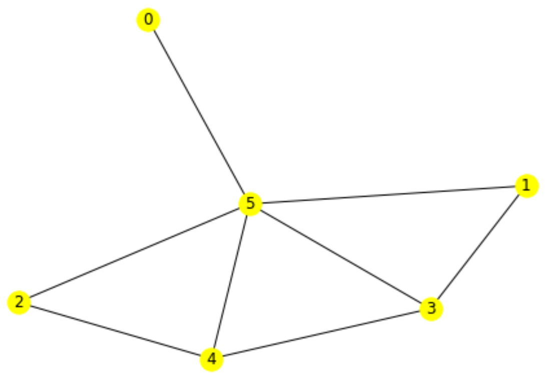

5421053822 - 0.9911

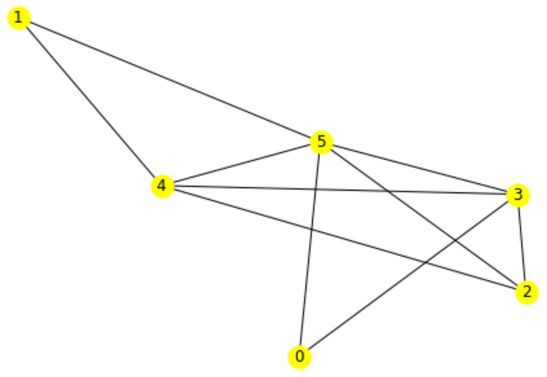

1092399998 - 0.9911

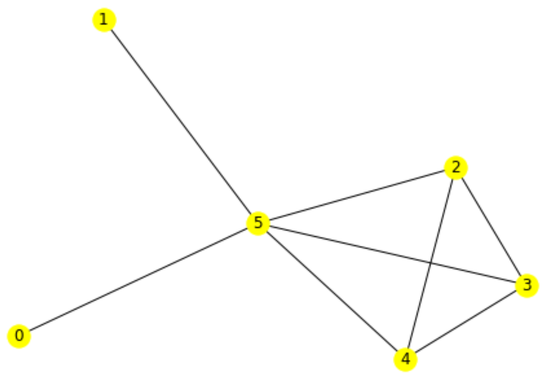

9723491702 - 0.9911

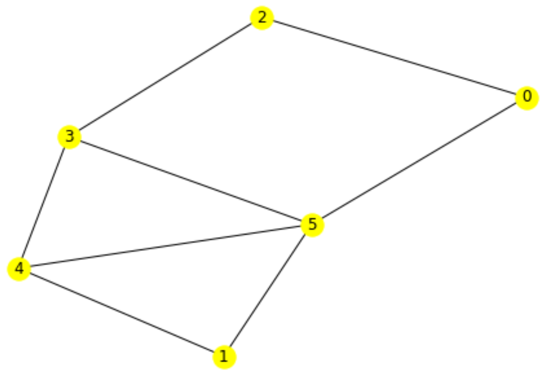

3340615550 - 3340615550

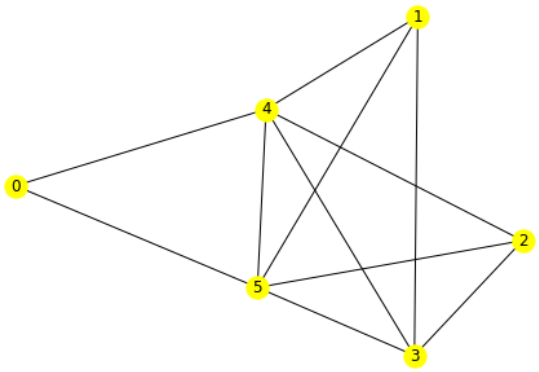

5419972478 – 1

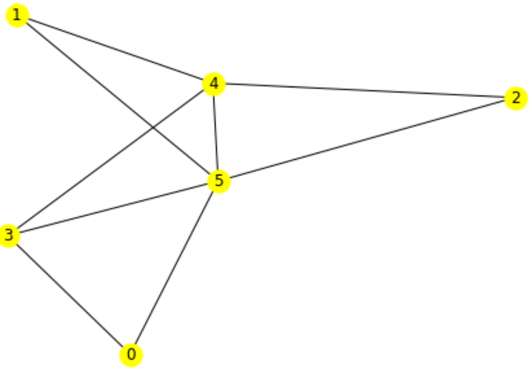

5421045374 – 1

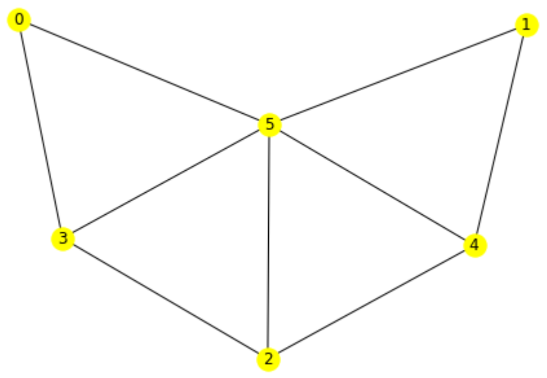

19882883934 - 1

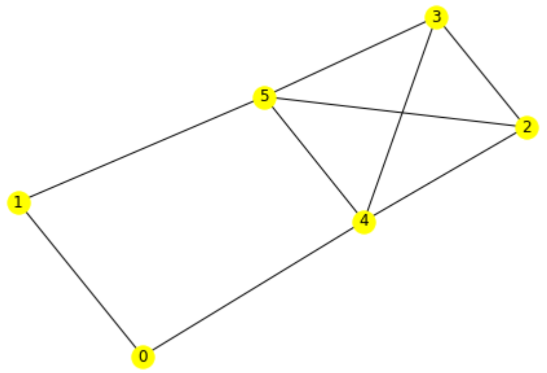

12945364566 - 0.9911

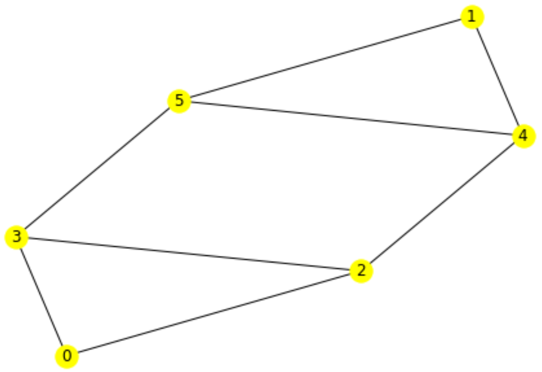

3272907836 - 0.9641

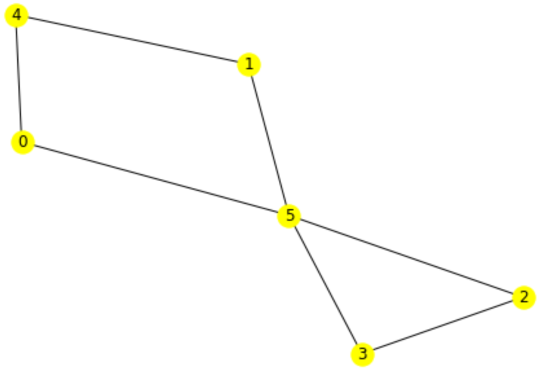

3273441150 – 0.9911

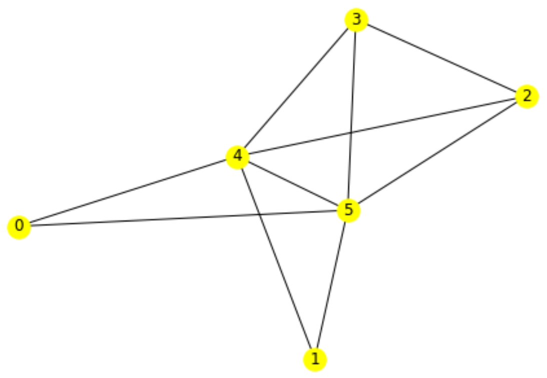

4467340622 - 0.9641

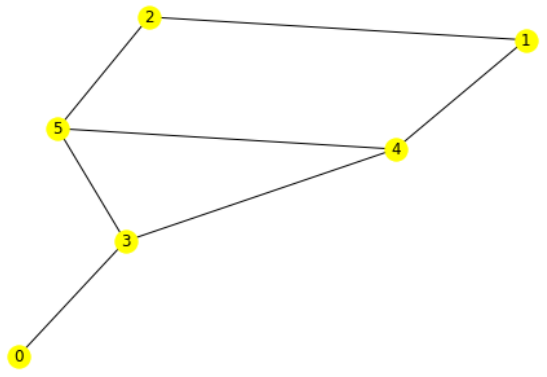

2266865246 – 1

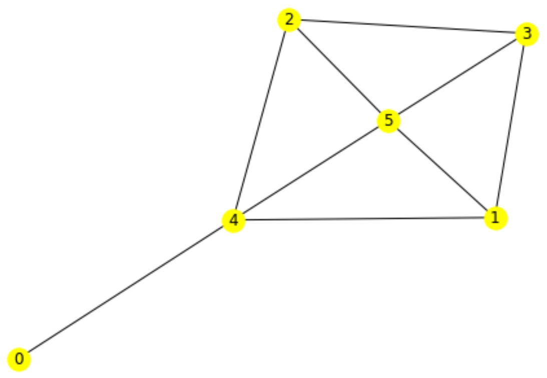
2
3
5
4
1
0

26348893518 - 0.9641

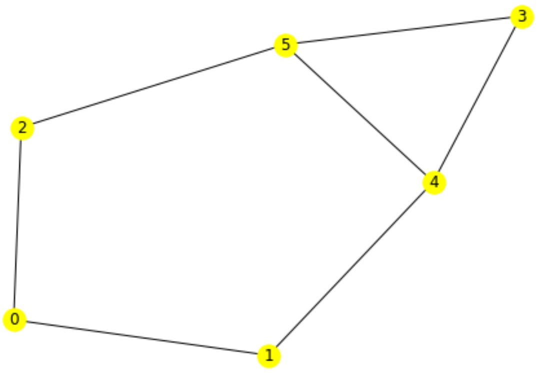

23137793918 - 0.9641

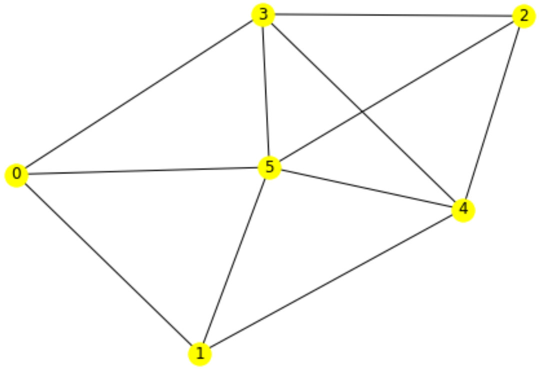

1125955388 - 0.9911

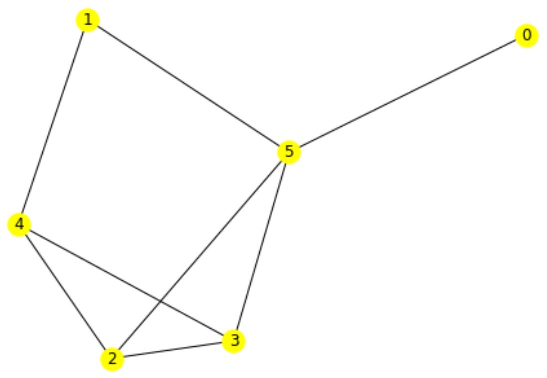

18842695484 - 0.9911

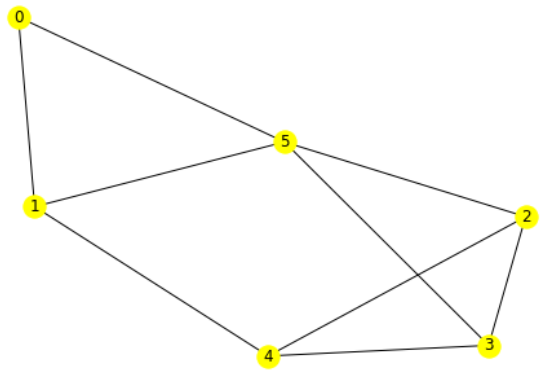

22064052062 - 0.7642

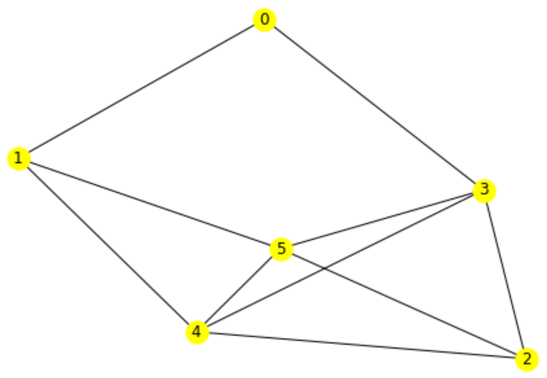

6665747294 - 0.9911

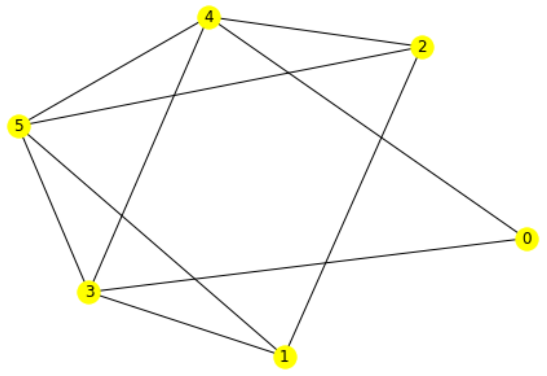

5420783222 - 0.9911

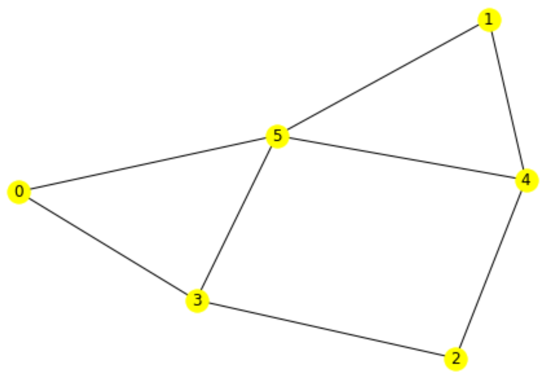

2165062494 - 0.9641

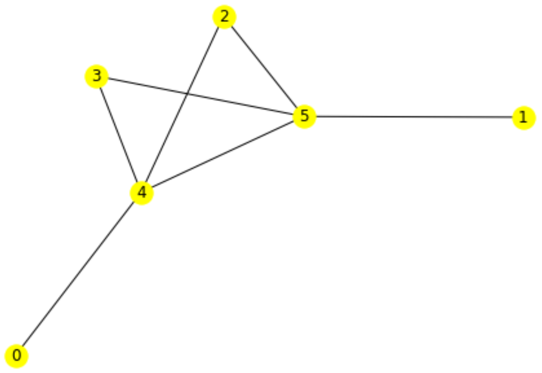

[

3427613550 - 1

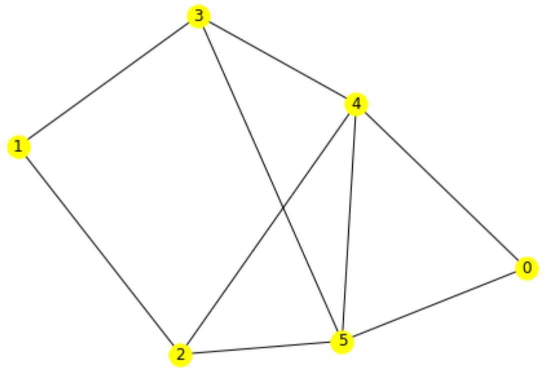

17923126094 - 0.9911

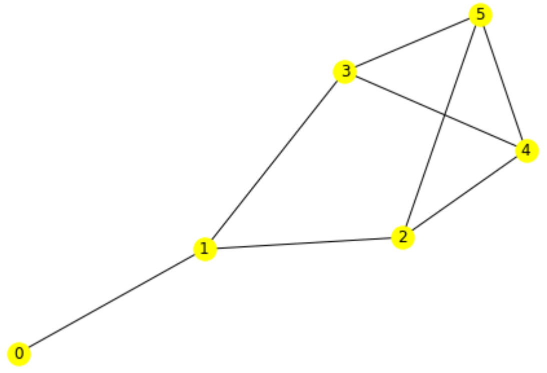

26399814494 - 1

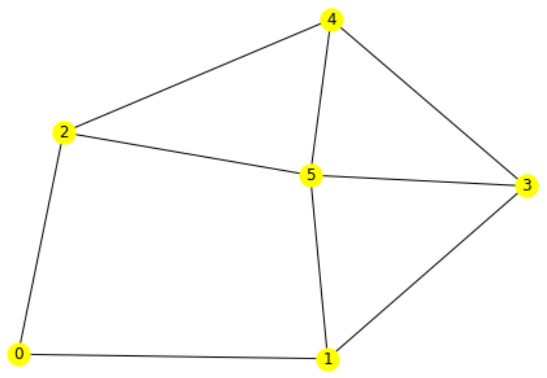

19881802590 - 0.9911

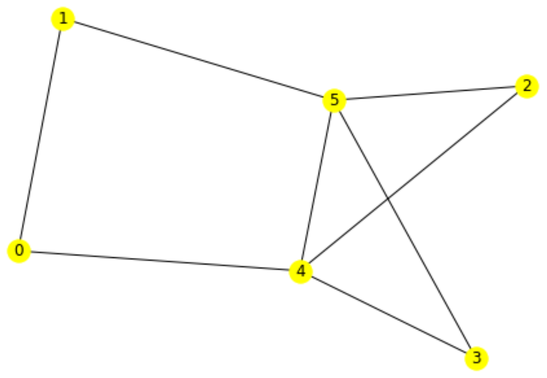

3340607102 - 0.9911

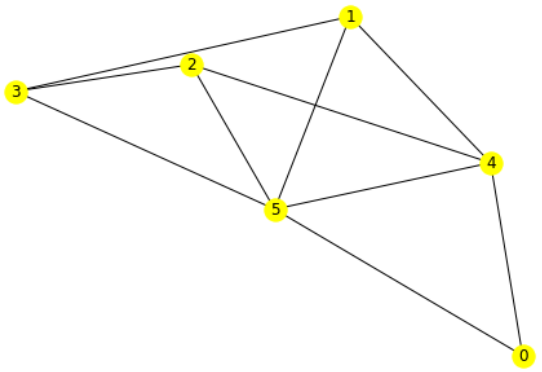

3394551150 - 1

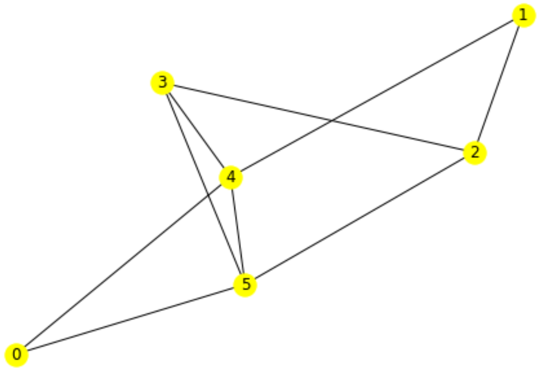

19882883868 - 0.9911

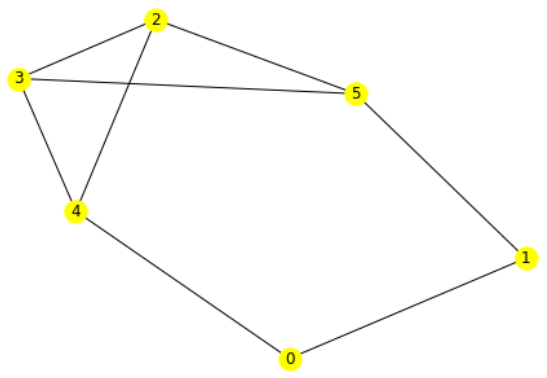

27508193150 - 0.9183

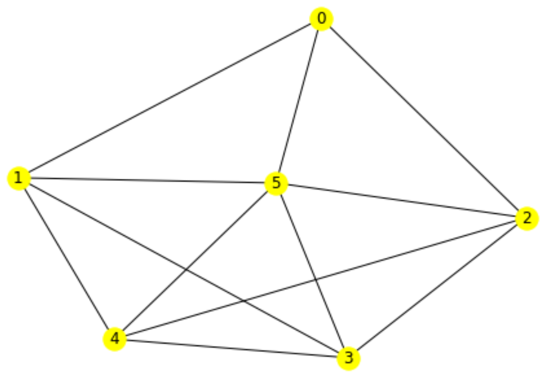

28547300190 - 0.9911

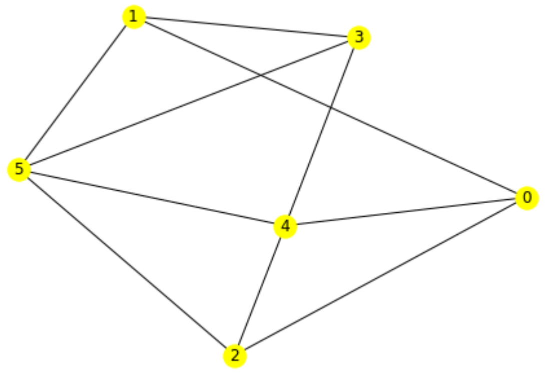

6527335262 - 1

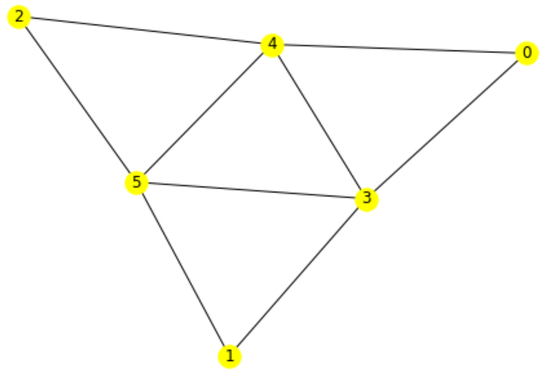

7635713918 - 0.9183

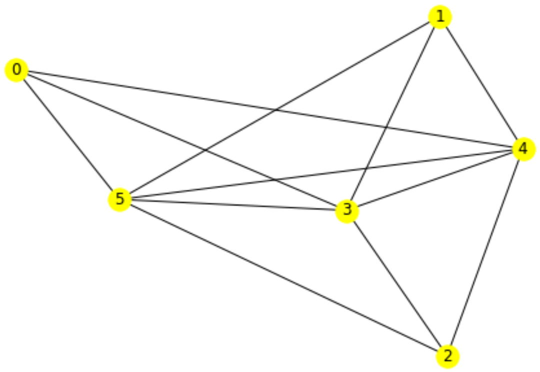

29655678846 - 0.8524

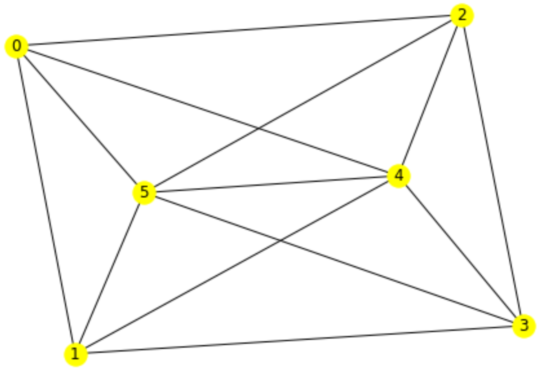

27473556350 - 0.9911

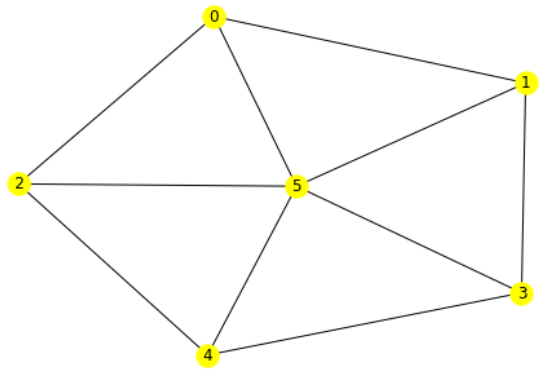

26434451294 - 0.9641

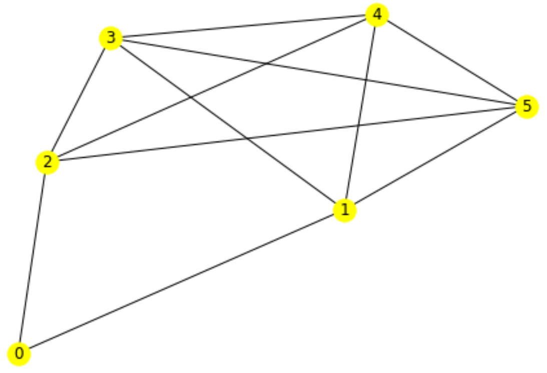

20990181180 - 0.9911

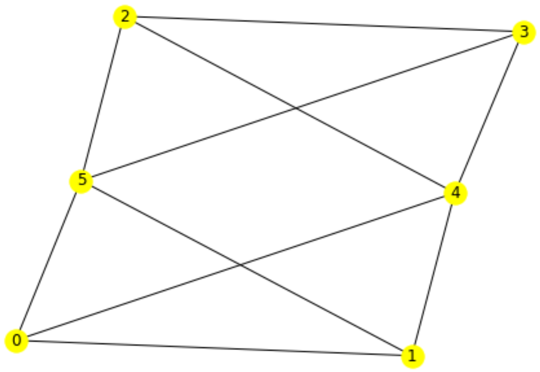

30782403918 - 1

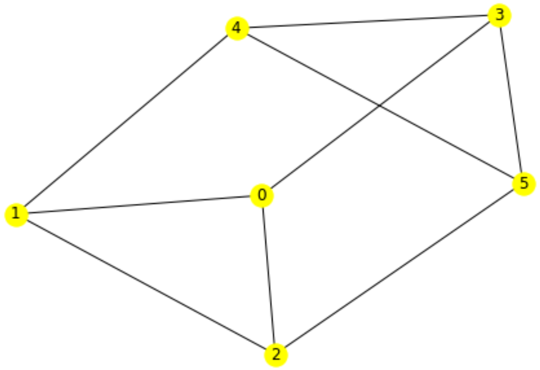

30662375262 - 0.9641

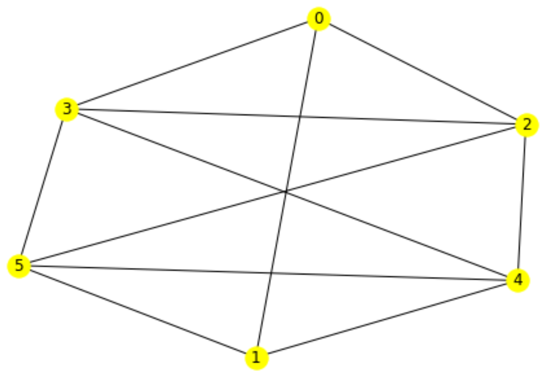

11938938684 -
